# New insights into the structural dynamics of the kinase JNK3

**DOI:** 10.1038/s41598-018-27867-3

**Published:** 2018-06-21

**Authors:** Pankaj Mishra, Stefan Günther

**Affiliations:** Institute of Pharmaceutical Sciences, Research Group Pharmaceutical Bioinformatics, Albert-Ludwigs-University Freiburg, Hermann-Herder-Straße 9, 79104 Freiburg, Germany

## Abstract

In this work, we study the dynamics and the energetics of the all-atom structure of a neuronal-specific serine/threonine kinase c-Jun N-terminal kinase 3 (JNK3) in three states: unphosphorylated, phosphorylated, and ATP-bound phosphorylated. A series of 2 µs atomistic simulations followed by a conformational landscape mapping and a principal component analysis supports the mechanistic understanding of the JNK3 inactivation/activation process and also indicates key structural intermediates. Our analysis reveals that the unphosphorylated JNK3 undergoes the ‘open-to-closed’ movement *via* a two-step mechanism. Furthermore, the phosphorylation and ATP-binding allow the JNK3 kinase to attain a fully active conformation. JNK3 is a widely studied target for small-drugs used to treat a variety of neurological disorders. We believe that the mechanistic understanding of the large-conformational changes upon the activation of JNK3 will aid the development of novel targeted therapeutics.

## Introduction

Understanding of the conformational dynamics of kinases has been an area of tremendous interests in the last ten years^1–6^. In recent years, the atomistic simulations have also helped to design several kinase inhibitors by unraveling the conformational rearrangements of the protein^7–11^. Here, we have studied the structural dynamics of the kinase known as c-Jun NH_2_-terminal kinase 3 (JNK3).

JNKs are serine/threonine kinases belonging to the evolutionary conserved mitogen-activated protein kinase (MAPK) family. JNKs are also known as stress-activated protein kinases because of their activation by extracellular stress stimuli and several cytokines. The JNK family members are of ubiquitous importance in regulating the response to stresses of diverse nature such as UV radiation, genotoxic, osmotic, hypoxic and oxidative stress^12–14^. Principally, *jnk1*, *jnk2*, and *jnk3* encode for three predominant isoforms viz. JNK1, JNK2, and JNK3^15–18^. All JNK proteins share a common protein kinase domain similar to other eukaryotic serine/threonine protein kinases. JNK1 and JNK2 are colocalized in most of the cell types while JNK3 is selectively expressed in the neuronal cells^15–17,19^.

Due to the preferential location of JNK3 in neuronal cells, it is a widely studied target for small-drugs used to treat a variety of neurological disorders such as Alzheimer’s disease^20^, Parkinson’s disease^21^, Huntington’s disease^22^ and Amyotrophic lateral sclerosis^23^. A goal of current research is to develop more selective inhibitors of JNK3^24,25^.

Dual phosphorylation of threonine and tyrosine residues of the conserved Thr-Pro-Tyr (TPY) motif (at the phosphorylation lip, also known as activation loop or A-loop) by the specific kinases MKK4 and MKK7 activates JNKs^12,14^. Activated JNKs then phosphorylate several nuclear and non-nuclear substrates such as c-Jun, ATF-2, Elk-1, the mitochondrial Bcl2 protein family, and others^26,27^.

It is assumed that the unphosphorylated state of JNK3 is found in the open conformation whereas the structural conformation of the phosphorylated JNK3, or JNKs in general, could not be elucidated yet. Several studies are known highlighting the allosteric regulation mechanism of peptide binding to JNKs^28–31^. However, little experimental evidence is available which can explain the underlying regulatory mechanism of JNK3 (or JNKs) in unphosphorylated and phosphorylated states.

**Figure 1:**
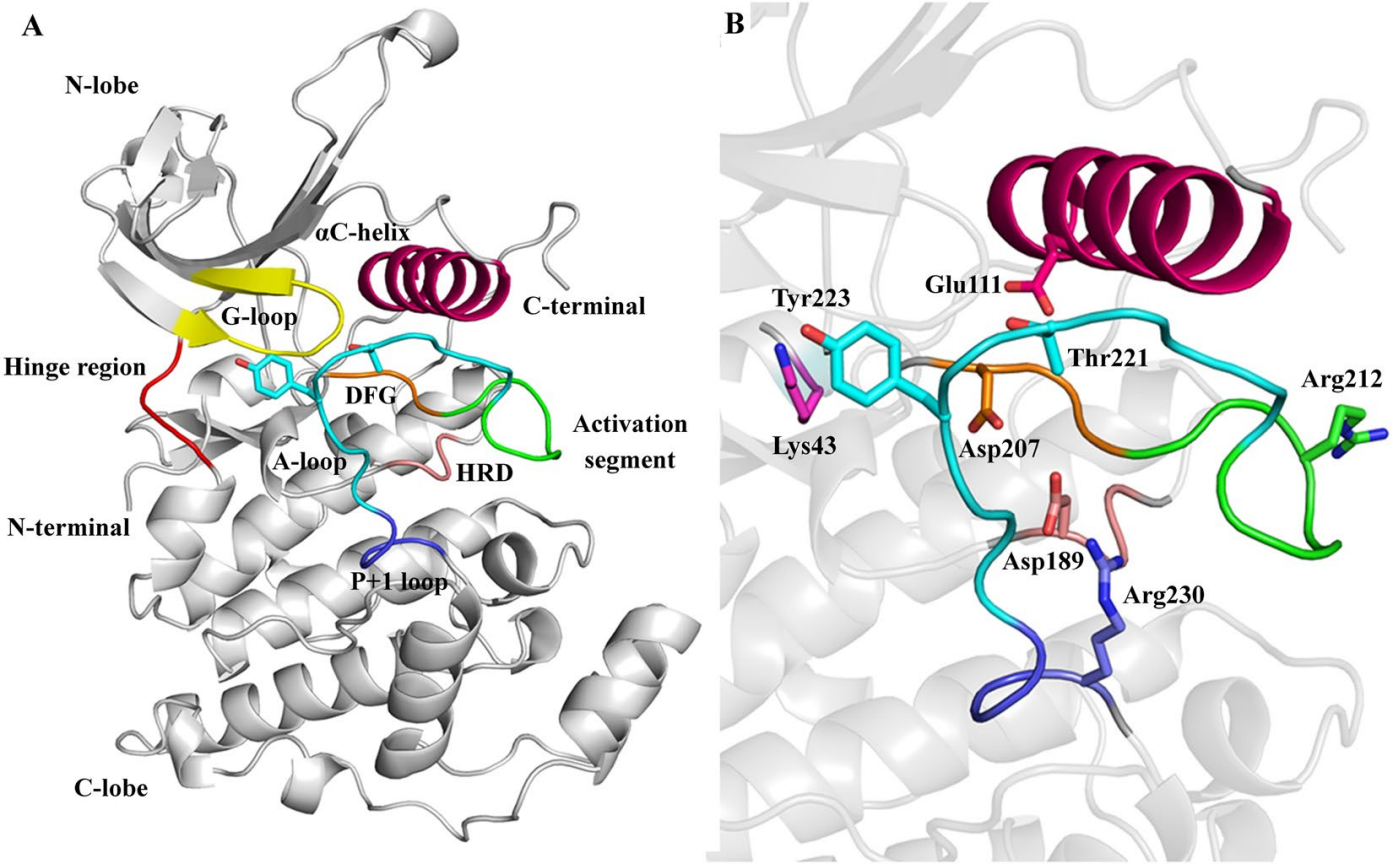
Three-dimensional structure of unphosphorylated JNK3 in the open state. (**A**) Classical bilobal kinase structure. Key structural elements are colored in yellow (G-loop), red (hinge region), pink (αC-helix), green (activation segment), orange (DFG motif), cyan (A-loop), purple (P + 1 loop), salmon (HRD motif) and gray (N- and C-lobe). (**B**) The key residues are shown in the sticks. Three-dimensional model of ATP-bound phosphorylated JNK3 is shown in Fig. S1.

Furthermore, no crystal structures are currently available describing the full structure of JNK3. The first 39 residues in N-terminal and last 62 residues in C-terminal are missing in the available crystal coordinates. It is also known that these regions are highly flexible and interfere with the lattice formation during the crystallization steps^32^. The structural organization of human unphosphorylated JNK3 is reported in Fig. 1.

JNK3 exhibits the typical architecture of a kinase containing the classical bilobal fold. The smaller N-terminal lobe is mainly composed of β-strands and one α-helix (known as αC-helix). The larger C-terminal lobe is predominantly α-helical and connected to the N-terminal lobe by a flexible hinge-like structure. The interface of the two lobes exhibits a deep cleft that characterizes the ATP-binding pocket and is in the closed state fully covered by a conserved glycine-rich sequence (G-loop, residues 71 to 78). Like other kinases, JNK3 also has the well-characterized structural motifs at the active site; the activation loop (A-loop, residues 217 to 226), the Asp-Phe-Gly (DFG, residues 207 to 209) motif and the αC-helix (residues 102 to 117).

The A-loop located at the C-terminal is the primary site of phosphorylation (Thr221 and Tyr223). It is the part of an extended activation segment, which begins at the DFG motif. The activation segment plays the most critical role in the regulation of the conformational changes in many kinases^33^.

In the C-lobe, also a highly conserved His-Arg-Asp (HRD, residues 187 to 189) motif plays a key role in the reaction catalysis^34^. Additionally, JNK3 also contains a D-recruiting site (DRS) which determines the interacting specificity of JNK3 to several cognate MAPKs (such as MKK4 and MKK7) and scaffold proteins such as JIP-1. The DRS is composed of three distinct motifs; the docking groove (residues 145 to 169), the ED site (residues 196 to 204) and a common docking domain (residues 359 to 372). The residues Arg227 to Arg230 belong to the P + 1 site and are deemed necessary for the binding of the peptide substrates. It is noteworthy that in unphosphorylated JNK3 the side-chain of Arg230 fills the P + 1 site in an unfavorable conformation for the binding of the peptide substrates.

The C-terminal domain also contains a MAP kinase insertion region located at the residues 283 to 328. In JNKs, the insertion region is 12 residues longer than its close homolog ERK1 and p38 leading to an extra 3_10_ helix and a αH helix extension of the N-terminus (Fig. S1)^32^.

In this study we envisaged unravelling the structural dynamics of the JNK3 kinase by studying the intrinsic conformational motions of three states *viz*. unphosphorylated JNK3 (uJNK3), phosphorylated JNK3 (pJNK3), and ATP-bound phosphorylated JNK3 (pJNK3-ATP) through MD simulation experiments. Additionally, to establish the relation between function and dynamics within the apo (uJNK3 and pJNK3) and ATP-bound (pJNK3-ATP) structures, we performed a principal component and free energy landscape analysis.

## Results

### Structural dynamics of apo and ATP-bound JNK3 kinase

Simulations of apo and ATP-bound JNK3 kinase were performed in this study. To fully understand the activation mechanism of the JNK3 kinase, it is important to study the conformational transitions imposed by the phosphorylation of the prototypical residues Thr221 and Tyr223. No structure is currently available for the phosphorylated JNK3 kinase. Moreover, the available structures also have several missing regions. In this study, we generated an all-atom structure of JNK3 from a high-resolution crystal structure by modeling the missing regions (see Figs S2 and S3). The modeled structure served as a starting point to generate the apo (uJNK3 and pJNK3) and ATP-bound (pJNK3-ATP) structures.

The atomistic MD simulations were used to study conformational transitions of the JNK3 kinase in these three states. The analysis of the raw trajectories from the 2 µs simulations data revealed a detailed conformational landscape of the transitions in the apo and ATP-bound structures.

Several studies indicated that the αC-helix could remain either in the αC-helix-in or in the αC-helix-out conformation and plays a crucial role in mediating the interplay between the open state to the closed state of different kinases^1,2^. In general, the helix αC contains a glutamic acid residue, which forms a salt bridge with the conserved lysine residue present at the β3 strands in N-terminal lobe and hence keeps the kinase in an open conformation. The lysine residues in turn coordinate with α- and β- phosphate groups of ATP^35^. The disruption of the salt-bridge interaction between the glutamic acid and lysine residue was reported as the initial step in the closure mechanism of many kinases^1^.

**Figure 2:**
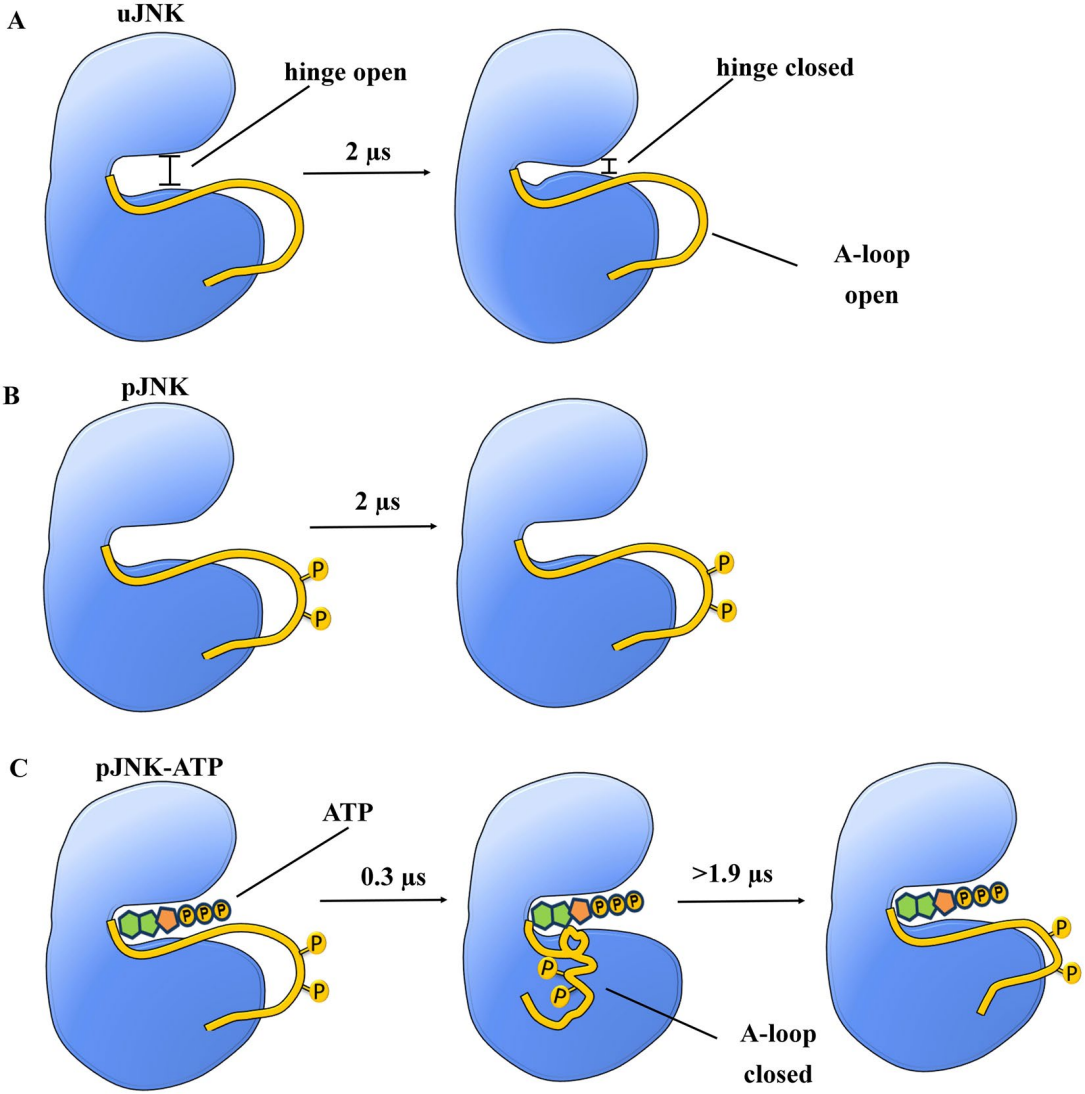
A schematic representation of kinase JNK3 cycling through different conformational states in the apo and ATP-bound structures. (**A**) In the native unphosphorylated JNK3 (uJNK3), both hinge and A-loop exists in an open conformation which at a higher time scale sequentially changes to a hinge-closed state whereas the A-loop remains in an open conformation. (**B**) Upon phosphorylation of the activation loop of native uJNK3 (represented by a yellow sphere; pJNK3), the hinge and A-loop stabilizes in its open conformation. (**C**) Upon binding of ATP to the phosphorylated JNK3 (pJNK3-ATP), the A-loop initially assumes a closed conformation and remains as such for a long duration and at a higher time scale it attains the open-like conformation whereas the hinge remains in an open conformation throughout the simulation period. The timescale mentioned in the scheme indicates the time in simulation at which the proteins attain the major transitions in this study.

The JNK3 used in this study is crystallized with adenylyl imidodiphosphate (AMP-PNP, an ATP analog) and two magnesium ions. The AMP-PNP molecule occupies the active site of JNK3 and forms a Mg^2+^ mediated interaction with the Glu111 of the αC-helix and maintains the open conformation of the protein. Our simulations indicate that the uJNK3 show a large-conformational transition from its native open conformation to the closed state whereas the pJNK3 and pJNK3-ATP retain the original open-like state. A scheme describing the different conformational states of JNK3 in the apo and ATP-bound structures during the 2 µs simulations is shown in Fig. 2.

**Figure 3:**
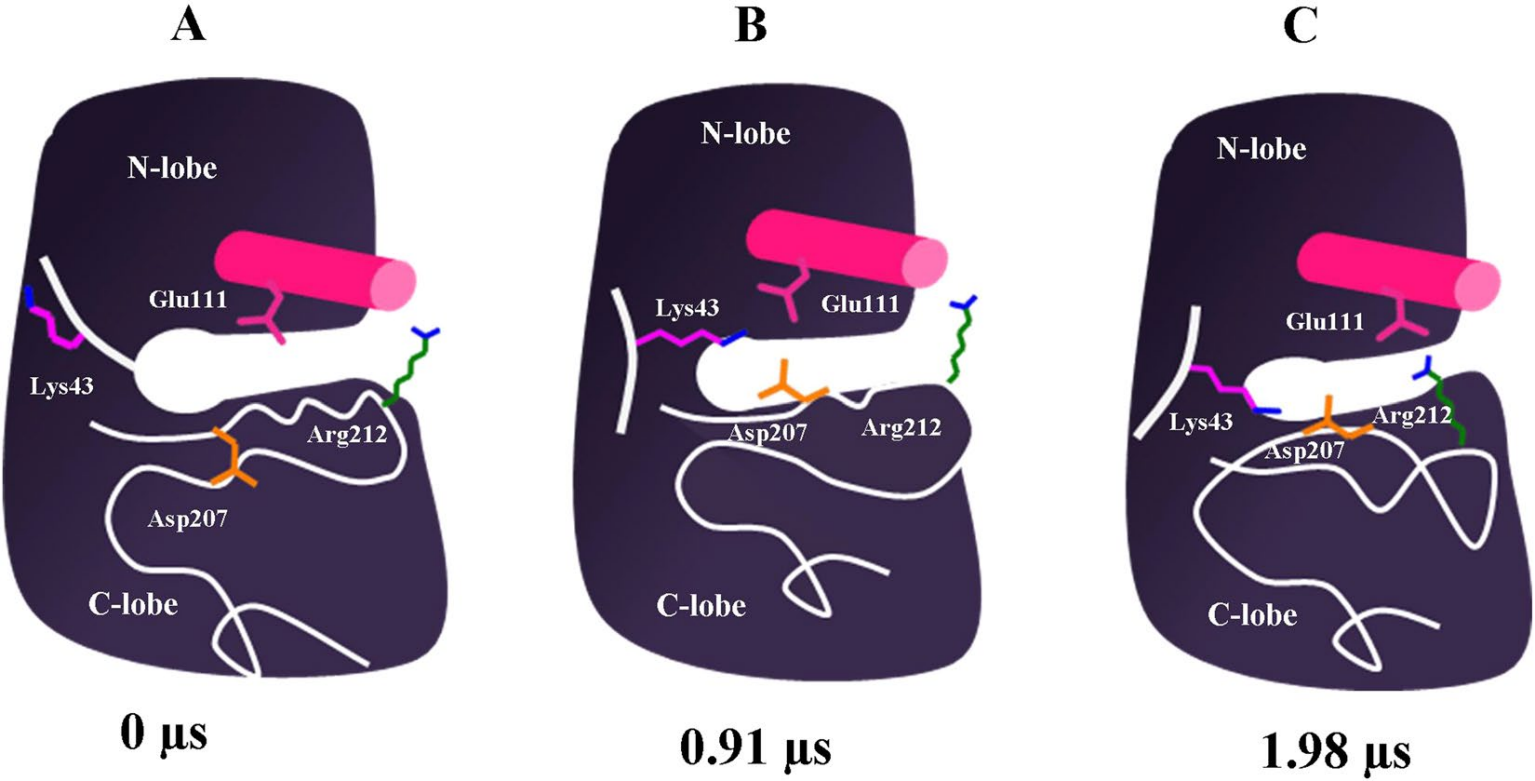
A schematic representation of the transition of unphosphorylated JNK3 from open to closed conformation.

**Figure 4:**
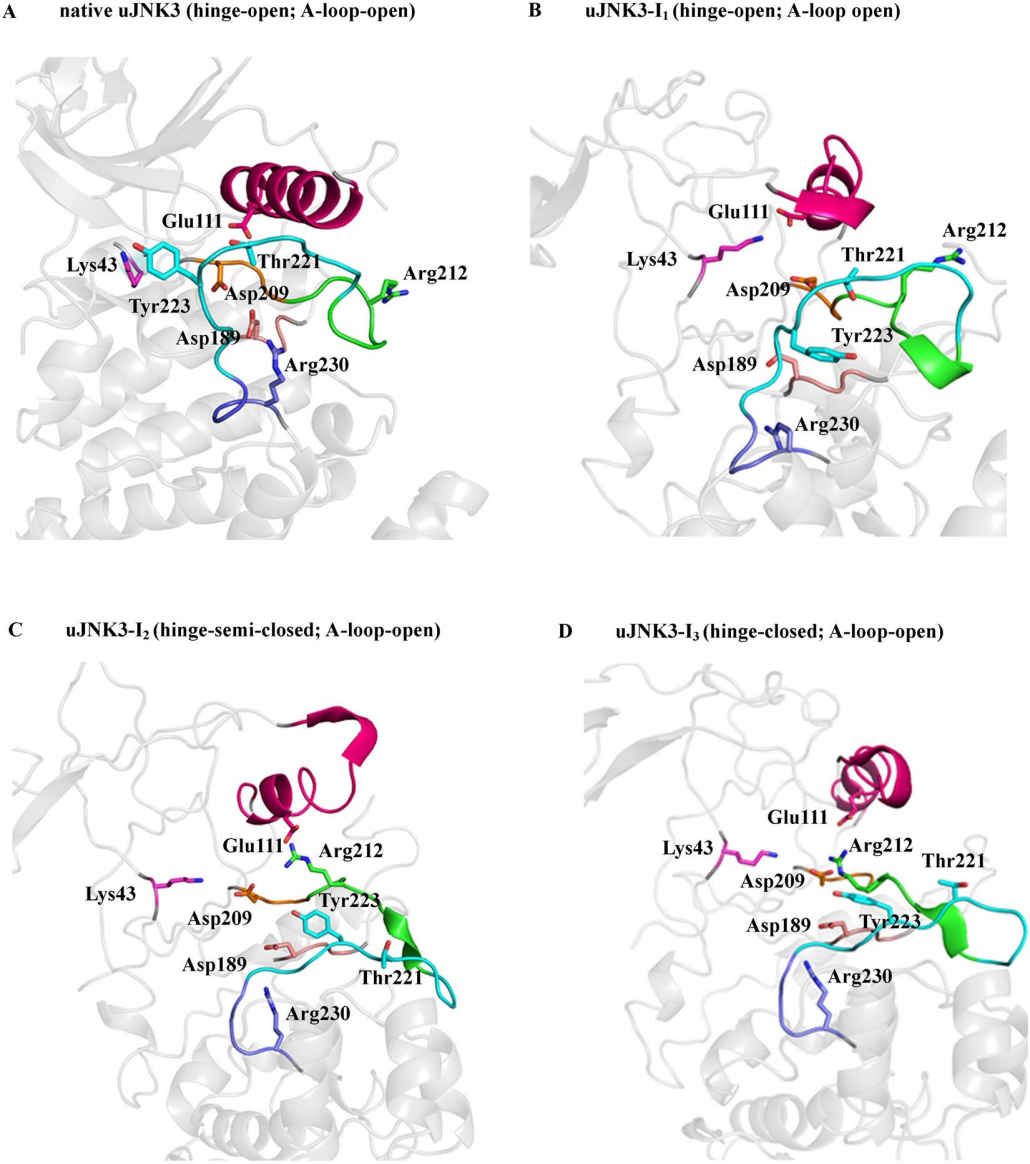
The conformational rearrangements of unphosphorylated JNK3 (uJNK3). **(A**–**D**) The uJNK3 adopted different conformations during its transitions from the native hinge-open to the hinge-closed state. The A-loop remains to stabilize in its open conformation. The highlighted areas are: pink (αC-helix), green (activation segment), orange (DFG motif), cyan (A-loop), purple (P + 1 loop), salmon (HRD motif) and gray (N- and C-lobe). The key residues are annotated in stick representation.

The open conformation is well-favoured initially in the unphosphorylated state. Moreover, during the course of the simulation, Lys43 re-orients itself (at ~75 ns) in such way that it forms a salt bridge interaction with Glu111 and anchor the αC-helix in the open conformation (uJNK3-I_1_, Figs 3B and 4B). The full closure of the unphosphorylated JNK3 occurs at a higher time scale (at ~1985 ns, uJNK3-I_3_, Figs 3C and 4D). However, to reach the closed state, the unphosphorylated JNK3 travels through an intermediate state (uJNK3-I_2_; Fig. 4C) as also indicated in the two-dimensional conformational landscape analysis (Fig. S4). This observation is broadly consistent with previous computational studies related to serine/threonine kinases as well as the tyrosine kinases^1,36–39^. The two-dimensional conformational landscapes were calculated as a function of the r.m.s.d of the A-loop and the difference of the distance between Glu111-Arg212 and Lys43-Glu111 residue pairs (see Fig. S7). The comparative analysis also helps to identify the major conformations that the pJNK3 and pJNK3-ATP occupy during the 2 µs simulation period. The two-dimensional conformational landscape and the intermediate protein structures of the pJNK3 and pJNK3-ATP are shown in Figs S5 and S6.

Broadly, the closure movement of unphosphorylated JNK3 can be summarized in two major steps as shown in Fig. 4. In the first step, the partial rotation (~45°) of αC-helix enables the breaking of the salt bridge interaction between Lys43 and Glu111 (uJNK3-I_2_, Fig. 4B). The breaking of this bond is favored by the proximity of the DFG motif. Arg212 approaching Glu111 initiates this step as it weakens the Lys43-Glu111 bond and allows the bond switch between Lys43-Glu111 and Lys43-Asp207. The DFG motif experiences out- to in-conformational movement which enables the Asp207 to form a salt bridge bond with Lys43. Notably, Asp189 of the HRD motif forms a hydrogen bond with Tyr223 and assists in the initial remodeling of the A-loop. Contrary to the observation made for the unphosphorylated CDK5 (another serine/threonine kinase) in which the Arg149 (Arg212 in JNK3) were observed to form a salt bridge with Glu51 (Glu111 in JNK3), in JNK3 Arg212 also forms a salt bridge with Asp207 for a short duration^1^.

To validate the reproducibility of the results and the observations made through the 2 µs simulations, two follow-up simulations were performed on a mutated and non-mutated uJNK3. In the non-mutated protein, the representative conformation obtained at step 2 (uJNK3-I_1_; Fig. 4B) was extracted from the initial trajectory and subjected to another simulation with similar conditions as defined for the original simulation. As expected, similar events were observed. The salt bridge change between Lys43-Glu111 and Lys43-Asp209 took place at a similar time-scale as in the original simulation as well as the outward rotation of the helix-αC has been initiated (see Movie S1). To validate the importance of this event for inducing the conformational change, a mutation experiment was performed on the same representative conformation (uJNK3-I_1_) as in the non-mutated system. Since Glu111 was clearly participating in the open-to-close movement of uJNK3, we have substituted Glu111 with alanine and performed the simulation again. A striking difference between the non-mutated and mutated structures of uJNK3 was observed at the DFG site; in former, the Asp209 quickly attain the in-orientation propelling the hydrogen bond switch from Lys43-Glu111 to Lys43-Asp209. By contrast, in the mutated uJNK3 the Asp209 mostly remains in the out-conformation and does not necessarily participate in forming a stable bond with the Lys43 (see Movie S2). Additionally, the interaction between the Ala111 and Arg212 could not be further observed due to the non-availability of the hydrogen bond acceptor in the Ala111 residue. Thus, the observations and interpretations of the native simulations can be considered as reproducible.

In the second step, the αC-helix adopts the out-conformation by rotating to ~90° and forms the strong salt bridge bonds with Arg212 (uJNK3-I_3_, Fig. 4D). The A-loop refold further to assume the closed conformation. Tyr240 and Glu242 stabilize the A-loop in this conformation by forming the hydrogen bonds with Tyr223. During the entire process, the salt bridge between Lys43 and Asp207 remains intact. Arg212 also interacts with Tyr223 via hydrophobic interaction.

In 1998 Xie *et al*. reported the first crystal structure of human JNK3 kinase^32^. They hypothesized the crucial role of basic residues *viz*. Arg188, Arg227, and Arg230 in the phosphorylation-related conformational changes of JNK3. Their hypothesis was based on the structure-based sequence alignment of human JNK3α1, human ERK2 and murine cAMP-dependent protein kinase (cAPK). Our study provides the first validation of the hypothesis proposed 20 years ago.

**Figure 5:**
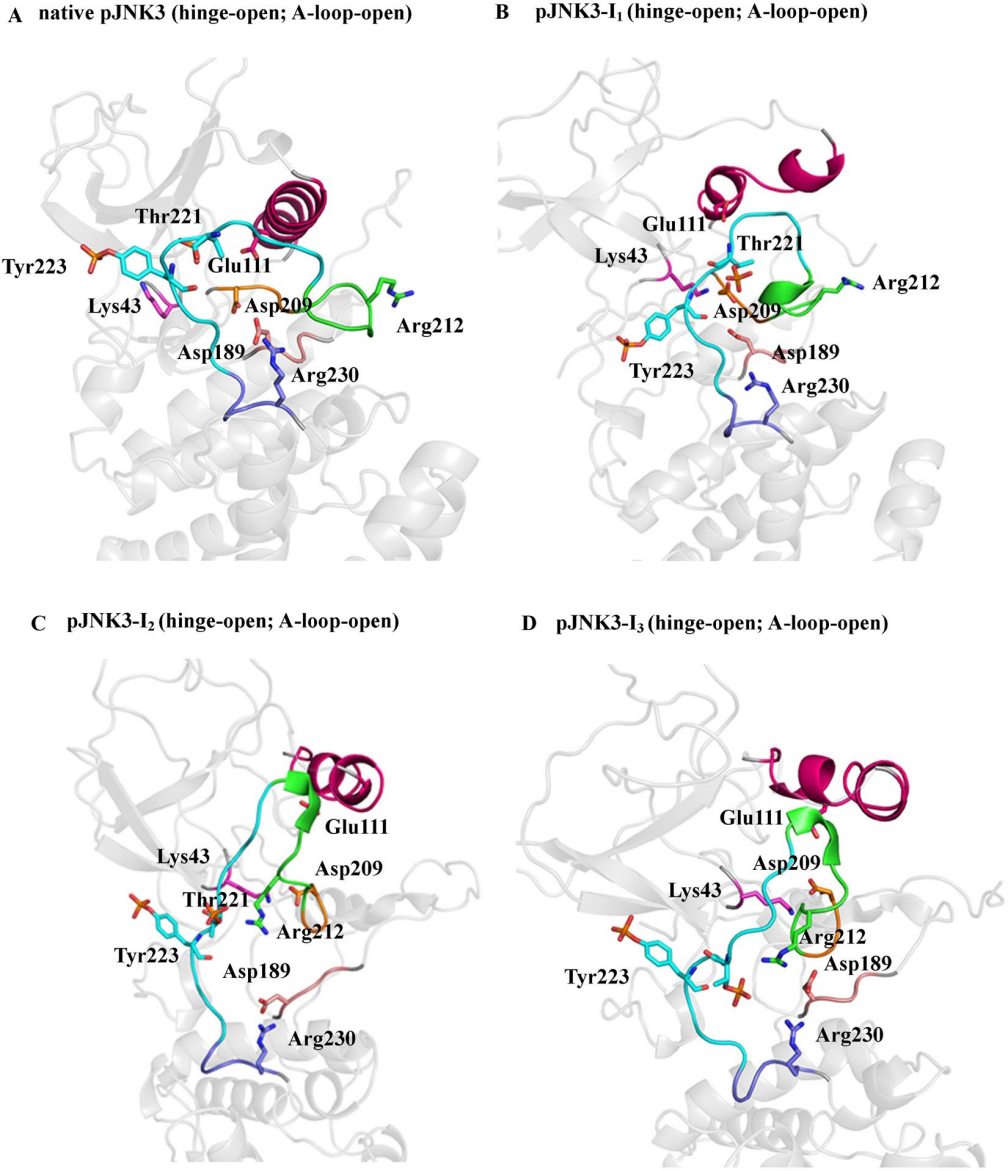
The conformational rearrangements of phosphorylated JNK3 (pJNK3). **(A**–**D**) The key conformations of pJNK3 during the simulation period are shown. The highlighted areas are: pink (αC-helix), green (activation segment), orange (DFG motif), cyan (A-loop), purple (P + 1 loop), salmon (HRD motif) and gray (N- and C-lobe). The key residues are annotated in sticks.

In uJNK3, we did not notice the basic residues playing any crucial role in the loop remodeling. However, in pJNK3 and pJNK3-ATP, the basic residues, particularly Arg227 and Arg230, contribute tremendously to the rearrangement of the A-loop. In both, the A-loop and hinge region remain in an open-like state initially. In pJNK3, the Lys43-Asp207 quickly (within ~3 ns) forms a strong salt-bridge (pJNK3-I_1_, Fig. 5B). A similar kind of bond was formed in the uJNK3 at a relatively high time scale. In the native state of JNK3, Arg230 exhibits a hydrogen bond with the Asp189. In uJNK3, the Arg230-Asp189 bond remains intact whereas in pJNK3 the bond switch takes place between Arg230-pThr221 and Asp189-Arg188. The making of the new bonds leads to the widening of the A-loop. Notably, Arg212 also forms the hydrogen bond with Asp189, and at a timescale of ~453 ns, the formation of the new bonds takes place between pThr221-Arg212, pThr221-Arg230, Asp189-Arg212, Asp189-Arg230, and Arg188-Asp189. The formation of the new bonds keep the A-loop and hinge region in an open-state (pJNK3-I_2_, Fig. 5C). At a higher time scale, the hydrogen bonds between pThr221 and Arg212 remains whereas Asp189 and Arg230 coordinate with each other (pJNK3-I_3_, Fig. 5D). The αC-helix remains stabilized in its native in-conformation.

**Figure 6:**
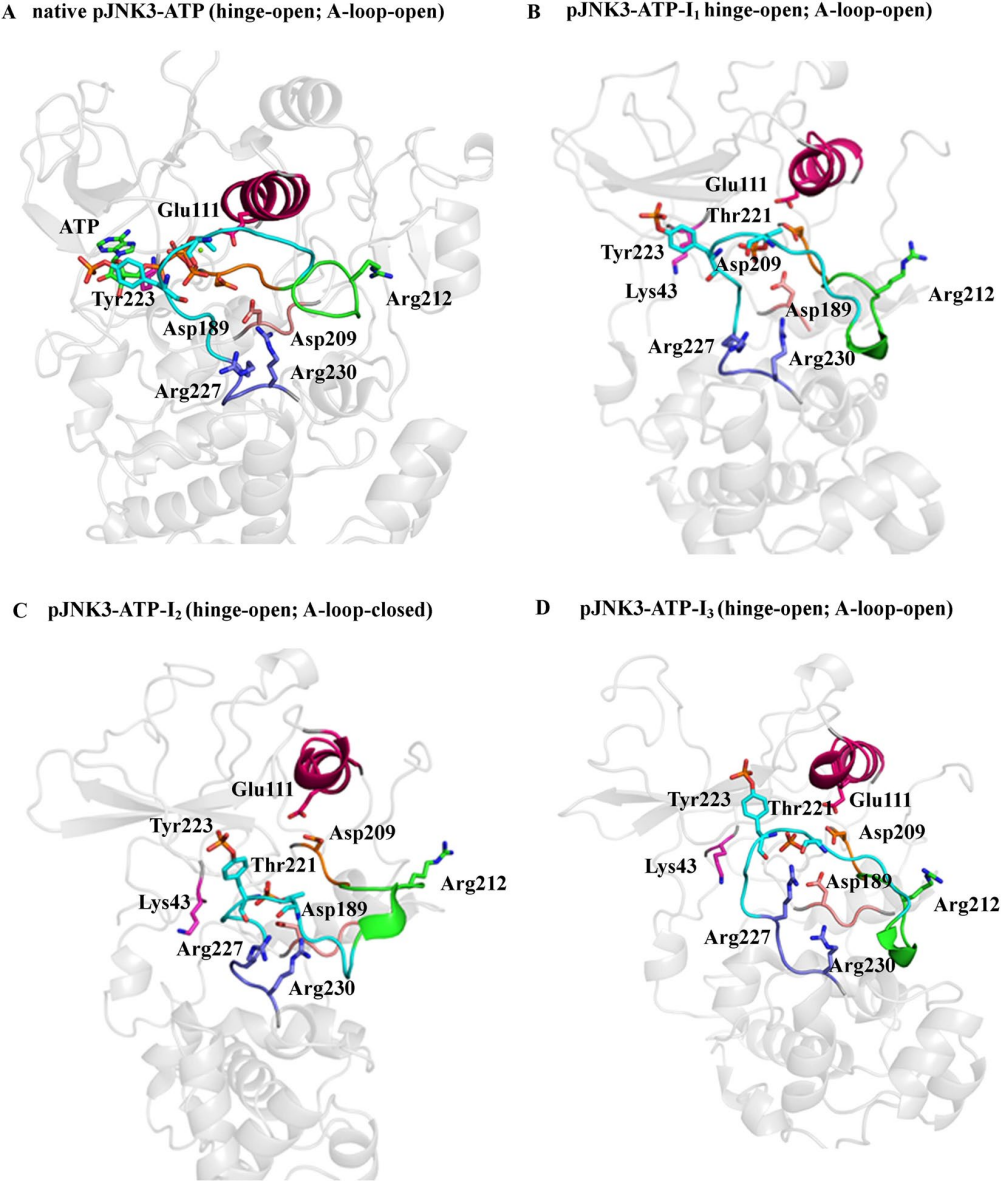
The conformational rearrangements of ATP-bound phosphorylated JNK3 (pJNK3-ATP). **(A**–**D**) The key conformations of pJNK3-ATP during the simulation period are shown. The highlighted areas are; pink (αC-helix), green (activation segment), orange (DFG motif), cyan (A-loop), purple (P + 1 loop), salmon (HRD motif) and gray (N- and C-lobe). The key residues are annotated in stick representations. The ATP molecule at the nucleotide binding site is shown in green sticks.

In pJNK3-ATP, the Glu111 and Asp207 coordinates to the ATP and Mg^2+^, pTh221 interacts with Arg227 via a hydrogen bond. The making of these coordination stabilizes the whole protein throughout the simulation period (pJNK3-ATP-I_1_, Fig. 6B). The native Asp189-Arg230 hydrogen bond remains to stabilize throughout the simulation. The pThr221 and Arg227 coordinate in such a way that the closure of A-loop takes place at the early stage of the simulation (pJNK3-ATP-I_2_, Fig. 6C) and remains intact for a long duration. However, at the late stage of the simulation period, the hinge assumes the open conformation as observed in the phosphorylated JNK3 (pJNK3-ATP-I_3_, Fig. 6D).

Xie *et al*. also mentioned that on phosphorylation Thr221 might interact with the basic residues *viz*. Arg227 and Arg188 leading to the closure of the A-loop, and Tyr223 might interact with the peptide substrate binding region residues (Arg227 and Arg230) forming new interactions in the phosphorylated, activated form.

The mechanism that we have found in the phosphorylated and ATP-bound phosphorylated JNK3 is closely related to the hypothesis provided by Xie *et al*. such that basic residues contribute to the conformational rearrangements of the A-loop. However, we did not notice pTyr223 making any contact with the basic residues in phosphorylated states. Moreover, the mechanism observed in our study supports the finding by Tournier *et al*.^40^. They reported that the phosphorylation of Thr221 alone could partially activate the JNK kinase with sufficient threshold. Since phosphorylated Thr221 preferentially coordinates with the basic residues such as Arg227, Arg230, and Arg212 (at the activation segment) and helps to keep the protein in the open state. Hence, the partial phosphorylation of the A-loop via Thr221 might activate the protein by allowing the partial but necessary conformational changes.

In Fig. S8 in the supporting information, the RMSD and RMSF during the 2 µs simulations are reported for the apo (uJNK3 and pJNK3) and pJNK3-ATP trajectories. In uJNK3 the closure movement displaced the G-loop completely making ATP binding impossible. Notably, most of the β-sheets arrangement in N-lobe evolves into the loop like structures. On the contrary, the pJNK3 and pJNK3-ATP preserve the integrity of their secondary structures in both N-lobe and C-lobe. Further, as expected, the addition of the intrinsically disordered residues at the N- and C-lobes and the phosphate groups at the A-loop altered the free energy minimum of the crystal structure.

### Conformational diversity of apo and ATP-bound JNK3 kinase

**Figure 7:**
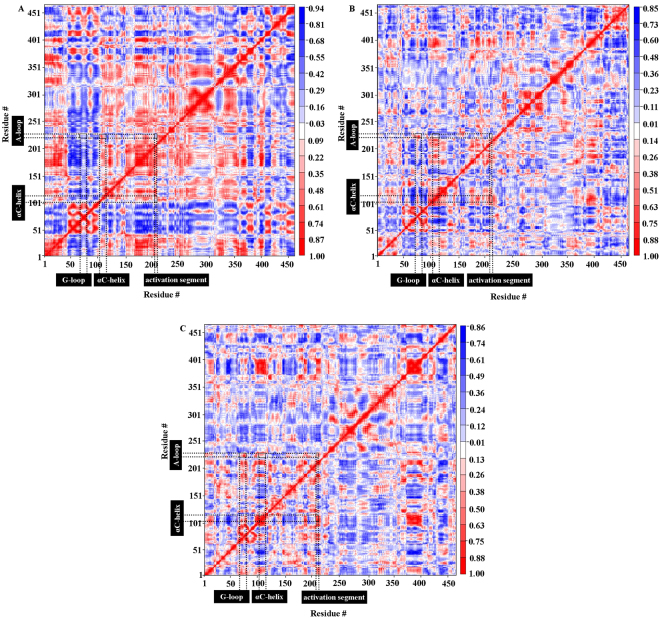
Cross-correlation matrixes of fluctuations of the JNK3 kinase. The cross-correlation matrixes were calculated as a function of Cα atom distance between residue pairs. The extent of correlated motions are shown using color-coded from red to blue (correlation to anti-correlation) for (**A**) uJNK3, (**B**) pJNK3, and (**C**) pJNK3-ATP.

A principal component-based cross-correlation analysis helped to probe the internal dynamics of the JNK3 kinase in unphosphorylated and phosphorylated states. The cross-correlation matrixes as a function of Cα atom distance between residue pairs are shown in Fig. 7.

The JNK3 kinase in the apo and ATP-bound states exhibit the obvious difference in their correlated motion. A visual inspection of the matrix shows that the different regions of uJNK3 are strongly correlated as shown in Fig. 7A (red coloured). For example, the key residues in the catalytic domain such as the His187-Asp189 (HRD motif), Glu111-Cys117 (αC-helix), Gly71-Val78(G-loop), and Arg227-Arg230 (P + 1 loop) are strongly correlated to the A-loop residues indicating a cooperative unfolding during the closure of the uJNK3 kinase. Moreover, the correlated motions in the pJNK3 and pJNK3-ATP are heavily reduced. In pJNK3, the key residues of the catalytic domain are anti-correlated (Fig. 7B; blue coloured) whereas, in the pJNK3-ATP (Fig. 7C), the His187-Asp189 (HRD motif), Glu111-Cys117 (αC-helix), Gly71-Val78 (G-loop), Lys210-Gly215 (activation segment) are very weakly correlated to the A-loop residues. Notably, in the pJNK3-ATP, the P + 1 loop (especially Arg227) is the only motif that strongly correlates to the A-loop residues.

We have analyzed the eigenvalues to quantitatively probe the conformational dynamics of the apo and ATP-bound JNK3 kinase. The eigenvalues obtained by the diagonalization of the covariance matrix of the Cα atomic fluctuations reveals that the few eigenvectors are sufficient to describe the concerted motions of apo and ATP-bound JNK3 kinase. Approximately, the first 35 eigenvectors describe the concerted motions of all the three trajectories. Collectively, these eigenvalues account for 88.1%, 77.6%, and 80.2% motions in uJNK3, pJNK3 and pJNK3-ATP, respectively. As shown in Fig. S12A, the degree of motions described by the first two eigenvectors are different. They capture more than 60%, 42%, and 43% of the total motion in uJNK3, pJNK3 and pJNK3-ATP, respectively. This result suggests that the internal motions of the JNK3 kinase in unphosphorylated (uJNK3) and phosphorylated (pJNK3 and pJNK3-ATP) states are limited to the essential subspace with fewer dimensions. Fig. S12B–D shows the projection of the MD trajectories onto the 1 and 2, 2 and 3, 30 and 35 eigenvectors. The first two eigenvalues indicate that all three trajectories reach distinct minima with small energy barriers. As evident from the Fig. S12A, the eigenvalues of uJNK3 are higher than the pJNK3 and pJNK3-ATP indicating that the uJNK3 experiences large conformational changes in its transition from the open state to the closed state.

**Figure 8:**
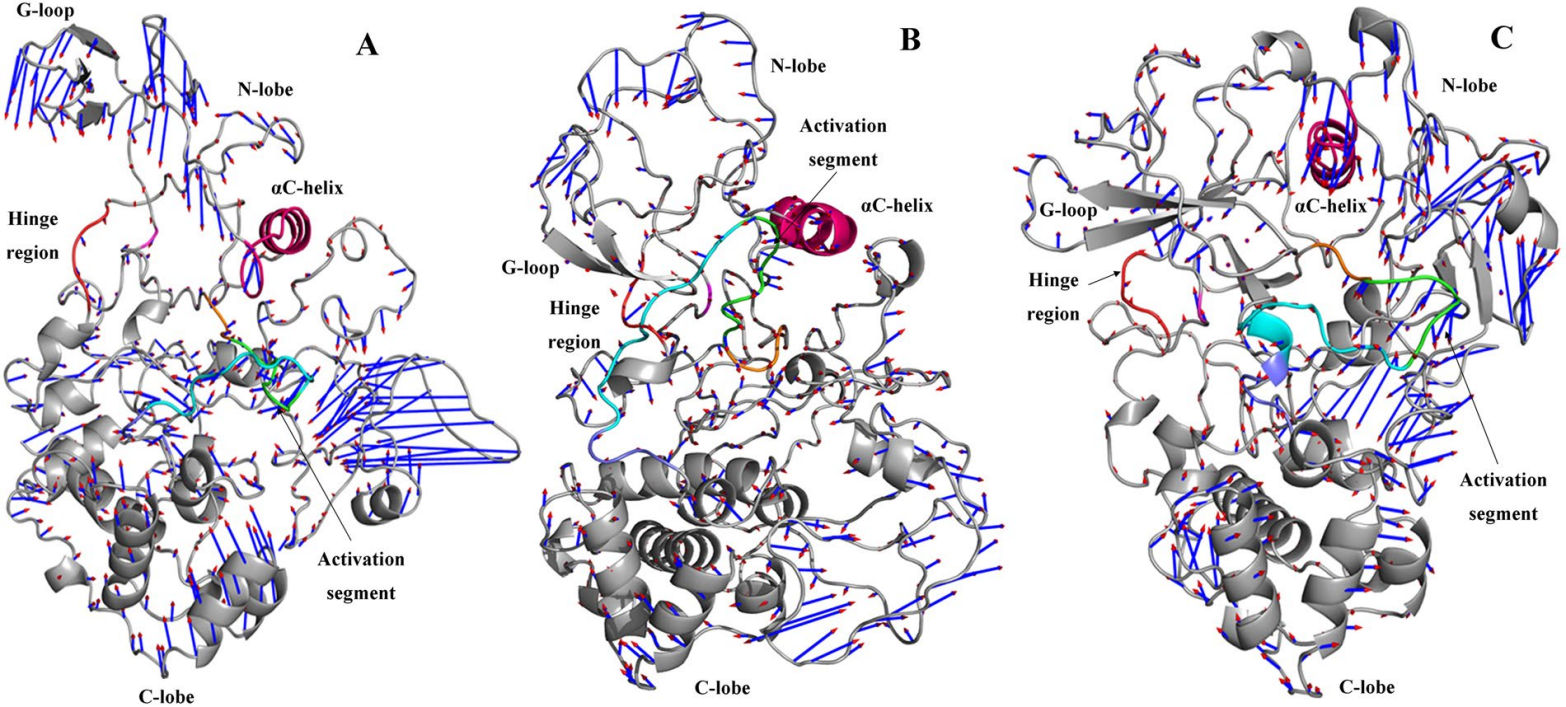
Porcupine plot of the first dominant motion mode. (**A**) uJNK3, (**B**) pJNK3, and (**C**) p JNK3-ATP. The blue arrow represents the direction of motion; the length characterizes the amplitude of motion. The highlighted areas are: pink (αC-helix), green (activation segment), orange (DFG motif), cyan (A-loop), purple (P + 1 loop), salmon (HRD motif) and gray (N- and C-lobe).

The subdomain motions captured by the first and second eigenvector of the three systems are shown in the Movies S3–S5 and S6–S8, respectively. The porcupine plot of the first dominant motion mode of the unphosphorylated (uJNK3) and phosphorylated (pJNK3 and pJNK3-ATP) JNK3 kinase is shown in Fig. 8.

As expected, the uJNK3 exhibits the largest domain motions as compared to the phosphorylated (pJNK3 and pJNK3-ATP) states. In uJNK3 (Fig. 8A), the N and C lobe moves towards each other. Notably, HRD motif, DFG motif, activation segment and A-loop collectively move towards the N-lobe with the largest motion in A-loop. The αC-helix and the G-loop move in an opposite direction and close each other. The P + 1 loop residues move away from the A-loop. Overall, the N and C-terminal intrinsically disordered residues (1–39 and 426 to 464) show the highest movements suggesting that they may play a role in propelling the dynamics of the other regions.

In pJNK3 (Fig. 8B), the N and C-lobe display different motion modes between them. The majority of the C-lobe helices move towards the N-lobe in the parallel mode. The αC-helix moves oblique upward to the catalytic domain residues from the C-lobe. The HRD motif, DFG motif, activation segment, A-loop and P + 1 loop collectively move towards the N-lobe. The pJNK3-ATP (Fig. 8C) exhibit the least pronounced motion in the catalytic domain. The N-lobe and αC-helix, and its adjoining loops collectively move towards the catalytic domain from the C-lobe. Moreover, the DFG motif and Glu111 (αC-helix) exhibits the constrained and more localized fluctuation which agrees well with changes observed in the analysis of the raw trajectories in the previous section.

## Discussion

JNK3 kinase plays a critical role in neurotoxicity^41–43^. It has also been implicated in the progression of many life-threatening neuronal and metabolic disorders. The majority of the drug discovery efforts for JNK3 have been directed towards targeting the ATP-binding site and inhibition of the interaction with the scaffold protein JIP1. Many inhibitors are known targeting these sites. However, due to the high similarity of the ATP-binding sites among the three JNK family members and the presence of the floppy regions (such as the nucleotide-binding loop, activation loop and the catalytic loop) which often experience a ligand-induced conformational change, it is difficult to target the ATP site of JNK3 specifically^44,45^. It is expected that targeting a specific conformation of the JNK3 kinase can help in the rational control of the induced conformational changes in the floppy regions^46^. The outcome would allow for the rational design of JNK3 kinase inhibitors that may exhibit higher affinity and address the selectivity issues by targeting the novel non-ATP competitive site. A similar approach has been discussed for other kinases. For example, Shukla *et al*. determined the activation pathway of the Src kinase, identified the intermediate states during transitions, and predicted the existence of binding pockets for novel allosteric inhibitors^3^. Furthermore, in an earlier publication by Knight *et al*., the authors suggested that targeting the allosteric site would help in the selective inhibition of kinases and would help to understand the function of the kinase in complex cellular signaling pathways^47^. The success of this strategy would be possible by identifying the key structural intermediates which may play a critical role in the underlying regulatory mechanism of the JNK3 kinase.

In this study, we have used the comparative molecular dynamics to investigate the intrinsic conformational motions of the JNK3 kinase in apo and ATP-bound states. A series of 2 µs MD simulations were performed on the unphosphorylated (uJNK3), phosphorylated (pJNK3) and ATP-bound phosphorylated JNK3 (pJNK3-ATP) kinase structures.

In the first step, we have reconstructed the full structure of the JNK3 kinase containing the intrinsically disordered regions present at the N-lobe and C-lobe. The molecular dynamics performed on the all-atom structure of JNK3 kinase helped to investigate the prime events and the key role of several known motifs. In the available crystal structures, the JNK3 exists in the unphosphorylated and open state. Moreover, the JNK3 used in this study is co-crystallized with adenylyl imidodiphosphate (AMP-PNP, an ATP analog) and two magnesium ions. The presence of the AMP-PNP and Mg^2+^ ions alter the native orientation of the ATP-binding pocket residues especially Asp207 (at the DFG motif), Lys43 and Glu111 (at the αC-helix). The altered residues re-orient themselves during the initial stages of the MD simulations and form salt-bridges. Strikingly, this event happens 25 times faster when JNK3 is phosphorylated (Figs 4B and 5B).

Our results also show that the uJNK3 undergoes the transition from the open state to the closed state. Overall, this transition takes place in two major steps mostly orchestrated by the catalytic domain residues. Firstly, the salt-bridge between Lys43 and Glu111 is broken, allowing the αC-helix to rotate at ~45°. This step is induced by the proximity of the Arg212 (a linker between the N- and C-lobes). A salt-bridge is formed between the Lys43 and Asp207. In this step, Arg212 also forms a temporary salt-bridge with Asp207. In the second step, the αC-helix is rotated by ~90° allowing the Glu111 to form a salt-bridge with Arg212. This step enables the transition of the N- and C-lobe into a closed orientation.

The pJNK3 and pJNK3-ATP do not undergo the conformational rearrangements at the hinge region and remain in the open-like conformation. However, in pJNK3-ATP, the rearrangement in the activation segment takes place allowing the refolding of the A-loop to the closed conformation which remains stable for a long duration of the 2 µs simulation period.

Further, the free energy landscape enabled the mapping of the atomistic path taken by the uJNK3, pJNK3, and pJNK3-ATP. Noticeably, the closed state of the uJNK3 appeared to be more stable than the open state. The associated free-energy profile also helped to identify the undisclosed low-energy intermediate conformations of the JNK3 kinase.

The behaviour of the kinase JNK3 observed through the MD simulations performed in this study was also reported in some other homologous kinases. In kinase CDK5, a large-scale movement was reported during the open-to-closed transition following a two-step mechanism similar to uJNK3^1^. A similar mechanism was also observed in the Abl kinase 1 and Src Kinase, in which the αC-helix, the DFG and activation loop residues coordinate in a similar concerted manner to attain the open-to-closed conformation^39,48^. Furthermore, Shaw and co-workers studied the “flip” from the DFG-in to the DFG-out state of the Abl kinase in a long timescale simulation (2.2 µs)^38^.

Meng & Roux observed that the phosphorylation of the A-loop residue Tyr416 of kinase c-Src stabilizes several structural features of the kinase c-Src and essentially locks the kinase into a catalytically competent conformation^49^. In a similar way we could observe that in the unphosphorylated JNK3 the A-loop showed a considerably higher flexibility compared to the phosphorylated structures (pJNK3 and pJNK3-ATP). In another kinase (PKA, protein kinase A), Hyeon *et al*. observed a ligand-induced transition upon binding of ATP, transforming the protein from open-to-closed conformation via a network of contacts^50^. Contrary to that, in phosphorylated JNK3, nucleotide-binding keeps the protein in an open state.

We expect that the identification of the key structural intermediates of the JNK3 kinase will support the rational design of allosteric JNK3 inhibitors. A druggability analysis of the different conformations of uJNK3 reavealed putative allosteric binding pockets that could be targeted by small molecules (Fig. S13). Nevertheless, through this work, we are also able to provide the mechanistic explanation of the two pioneer observations made by Xie *et al*.^32^ and Tournier *et al*.^40^. Herein, we validated the hypothesis of the Xie at al. in which they proposed the crucial role of the basic residues in the phosphorylation-related conformational changes of JNK3. Furthermore, we have provided the atomistic-level explanation of the finding by Tournier *et al*. addressing the preferential role of the phosphothreonine (at the A-loop) in the activation mechanism of the JNK3 kinase.

## Methods

### Structure preparation

No crystal structure of the phosphorylated (pJNK3) or ATP-bound phosphorylated JNK3 (pJNK3-ATP) is currently known. The available entries in the protein data bank have common missing residues from 1–44 and 401–464. Moreover, PDB entry 1JNK (resolution: 2.30 Å) is available as a complex with β, γ-imidoadenosine 5′-triphosphate (AMP-PNP) and two magnesium ions. Further, we selected the recently published PDB entry 4WHZ (resolution: 1.79 Å) complexed with the aminopyrazole derivative. The PDB entry 4WHZ also has the lowest number of the missing residues in between 45 to 464. The r.m.s.d between the 1JNK and 4WHZ is 0.55 Å. Hence, we used 4WHZ for further studies. The AMP-PNP molecule and the two magnesium ions were retained from the 1JNK. First, the missing residues 212 to 216 and 374 to 382 were added using the MODELLER program^51^. In the next step, we used the ab initio modeling protocol to generate the intrinsically disordered regions residues 1 to 44 and 401 to 464. The selection of the ab initio method was supported by the fact that the closest homologs of JNK3 such as JNK1 and JNK2 lack the initial 38 residues. We used the recombinant ab initio approach to determine the best model. The initial models were generated by using the GeneSilico metaserver^52^, Robetta^53^, Quark^54^, HHpred^55^ and Multicom server^56^. The outputs of these servers were then submitted to the QA-recombineIt server^57^. The best models were selected based on the ranking provided by the inbuilt ‘Model Quality Assessment Programs’. The obtained models were then added to the corrected crystal coordinate of the PDB entry 4WHZ using the MODELLER program. The high-ranked top three models were selected for the energy minimization step by Gromacs molecular dynamics package. The final model of the unphosphorylated JNK3 (uJNK3) kinase was selected based on the Ramachandran plot assessment^58^.

The Vienna-PTM 2.0 web server was used to phosphorylate Thr221 and Tyr223^59^. The ATP-molecule was docked onto the ATP-binding site of the pJNK3 using the Extra Precision (XP) module of the Glide (Schrödinger suite)^60^ while keeping the two magnesium ions from the PDB entry 1JNK.

### Molecular Dynamics Simulations

In this work, the distributed MD simulations were performed using GROMACS^61^ on the bwForCluster BinAC of the state Baden-Württemberg, Germany (http://www.binac.uni-tuebingen.de/). The GROMOS9643a1 force field and PRODRG2.5^62^ were used to generate the topology of the proteins and ATP, respectively. Each protein or complex was solvated in a cubic box of simple point charge water molecules. The boundary of the box was kept at least 12.0 Å away from any solute atom. The appropriate numbers of the counterions neutralized each system. Covalent bonds involving hydrogen atoms were constrained by applying the LINCS algorithm^63^. The long-range interactions were treated with the Particle Mesh Ewald (PME) method^64^. The Lennard-Jones potentials were used for Vander walls interactions^65^. The distance cut-off of 10.0 Å was applied to calculate the non-bond interactions. The prepared systems were subjected to the energy minimization applying the steepest descent algorithm. No restraint was applied during this step. Following the minimization steps, each system was equilibrated for one ns at constant temperature and pressure after applying position restraints to the protein. Finally, a 2 µs production phase was carried for each system. The temperature and pressure of each system were kept constant at 300 K and 1 bar, respectively. A 2 fs time step was applied.

### Principal component analysis

The internal collective motions of apo and ATP-bound JNK3 kinase were explored by analyzing the positional covariance matrix computed as the function of Cα atom distance between residue pairs and its eigenvectors. The Gromacs in-built tool ‘gmx covar’ was used to the extract the eigenvalues and eigenvectors of the MD trajectories extracted from each time frame of 50 ps MD trajectories. Prody plugin implemented in the VMD was used to analyze the collective motion of the apo and ATP-bound JNK3 kinase^66^. Another Gromacs in-built tool ‘gmx anaeig’ was used to analyze and plot the eigenvectors. Pymol python script ‘modevectors.py’ was used for porcupine plots graphics.

### Energy landscapes

The energy landscapes of the apo and ATP-bound JNK3 kinase were calculated as a function of the r.m.s.d of the A-loop and the difference of the distance between Glu111-Arg212 and Lys43-Glu111 residue pairs. The following equation was used to compute the free energy;

$${G}_{i}=-\,{k}_{B}Tln(\frac{{N}_{i}}{{N}_{max}})$$

where *G* is Gibbs free energy, *i* is bin, *k*_*B*_ is Boltzmann’s constant, T is the temperature of simulation system, *N*_*i*_ is the population of the bin and *N*_*max*_ is the population of the lowest free energy structure. In our calculations, the temperature were kept constant at 300 K. Gromacs in-built tool ‘gmx sham’ was used to estimate the free energy landscapes.

### Data interpretation and definition of states

The MD simulation is described and interpreted by measuring the RMSD and RMSF. The principal component analysis and free energy landscape were performed by using the Gromacs in-built tool. The ligand pocket detection was performed by using Fpocket^67^. Three states viz. open, intermediate and closed were considered to analyze the conformational transformations of kinase JNK3 during the MD simulations. The open conformation of kinase JNK3 was obtained from the protein databank (pdb id: 4WHZ). In the open conformation, the hinge region between the N- and C-lobe remains wide open with Glu111 and Arg212 as the gatekeeper residues lying far from each other. The closed conformation is the representative structure in which the Glu111 and Arg212 interact to attain the closure of the hinge region. The intermediate states are the major conformations in between the transition of the kinase JNK3 from the open-to-closed conformation during the 2 µs.

## Electronic supplementary material

Supplementary Information

Movie S1

Movie S2

Movie S3

Movie S4

Movie S5

Movie S6

Movie S7

Movie S8
