# Supplementary material for: New insights into the structural dynamics of the kinase JNK3
Source: Sci Rep. 2018 Jun 21;8:9435. doi: 10.1038/s41598-018-27867-3 (PMC6013471; doi:10.1038/s41598-018-27867-3)
Supplement: Supplementary file 1 — Supplementary Information [file 41598_2018_27867_MOESM1_ESM.pdf]

Supplementary Information

New insights into the structural dynamics of the kinase JNK3

Pankaj Mishra[1], Stefan Günther[1*]

1. Institute of Pharmaceutical Sciences, Research Group Pharmaceutical Bioinformatics, Albert-Ludwigs-University Freiburg, Hermann-Herder-Straße 9, 79104 Freiburg, Germany.

E-mail address: stefan.guenther@pharmazie.uni-freiburg.de

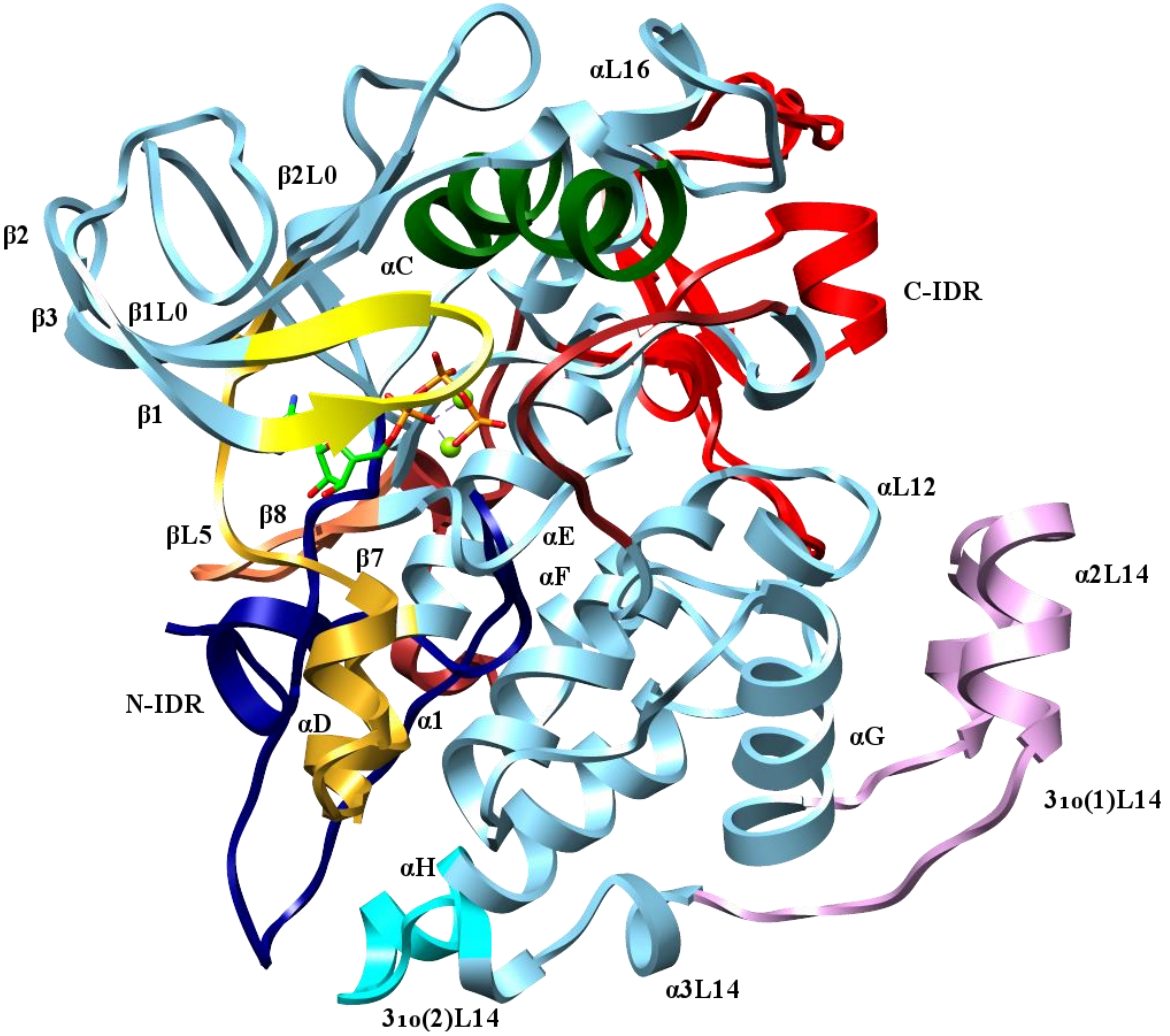

Figure S1. Three-dimensional structure of ATP-bound phosphorylated JNK3 (pJNK3-ATP). Structural elements are colored in blue (N-terminal intrinsic disordered region), red (C-terminal intrinsic disordered region), yellow (G-loop), dark red (αC-helix), firebrick red (A-loop), cyan (insertion residues), sandy brown (αC-helix hinge), golden (docking groove), coral (ED domain), dark brown (CD domain) and pink (extra 3_{10} helix region). The ATP molecule at the nucleotide binding site is shown in green stick representation. Phosphorylated residues (Thr221 and Tyr223) are also shown in the stick representations. The structural assignments followed the crystal structure presented by Xie et al[1].

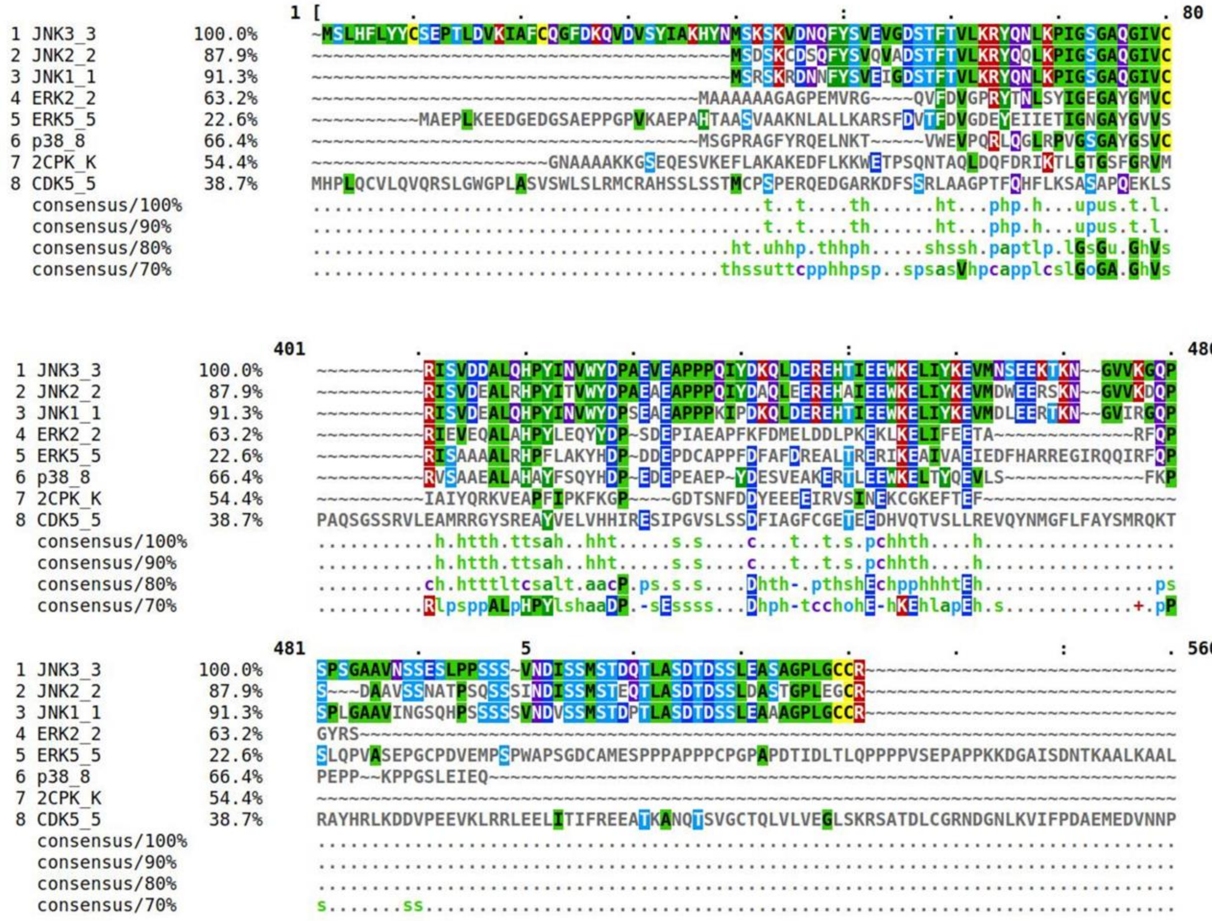

Figure S2. Multiple sequence alignments of the intrinsically disordered regions of JNK3 with other homologous kinases *viz.* ERK2, ERK5, human p38, cAPK (pdb id: 2CPK) and CDK5.

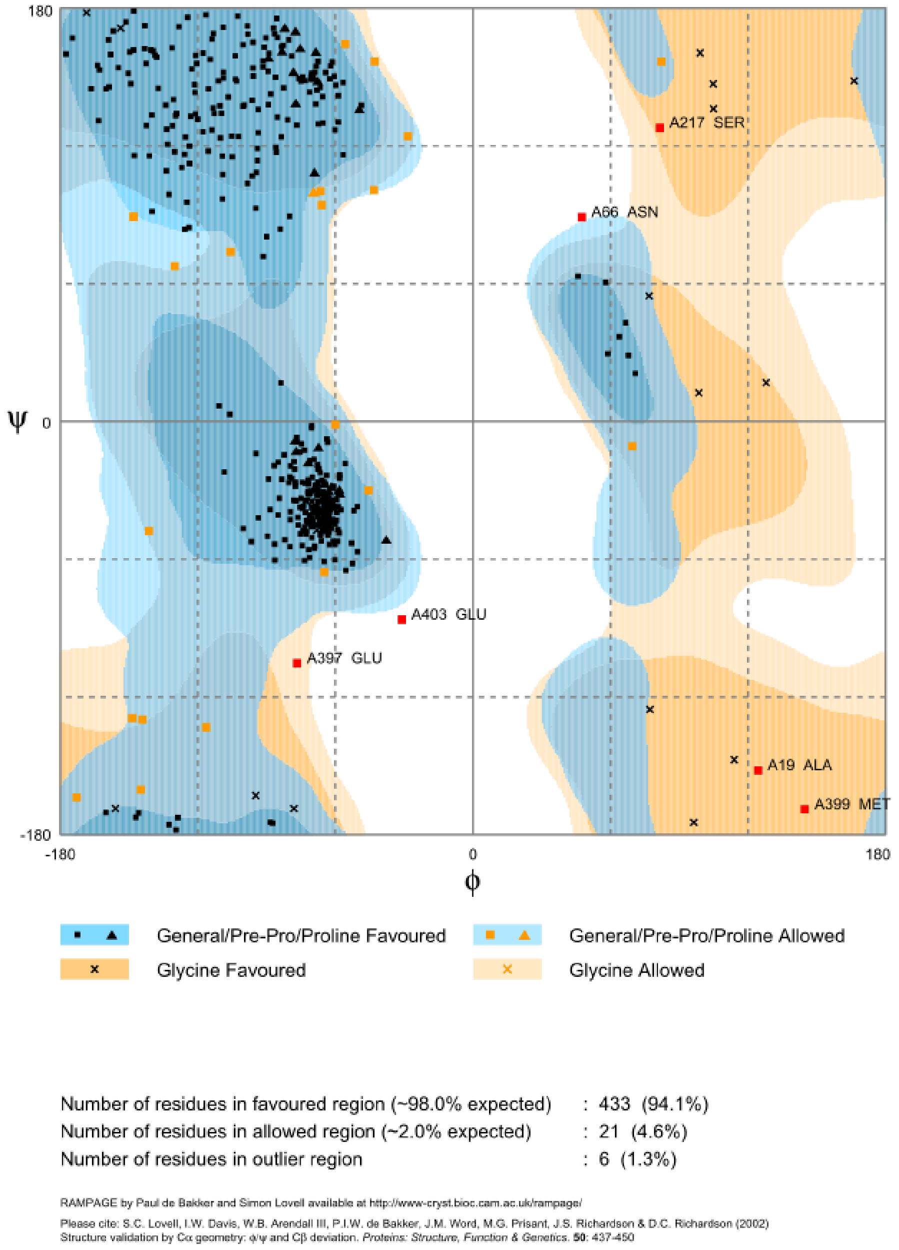

Figure S3. Ramachandran plot of the best model of JNK3 selected for the MD simulations studies.

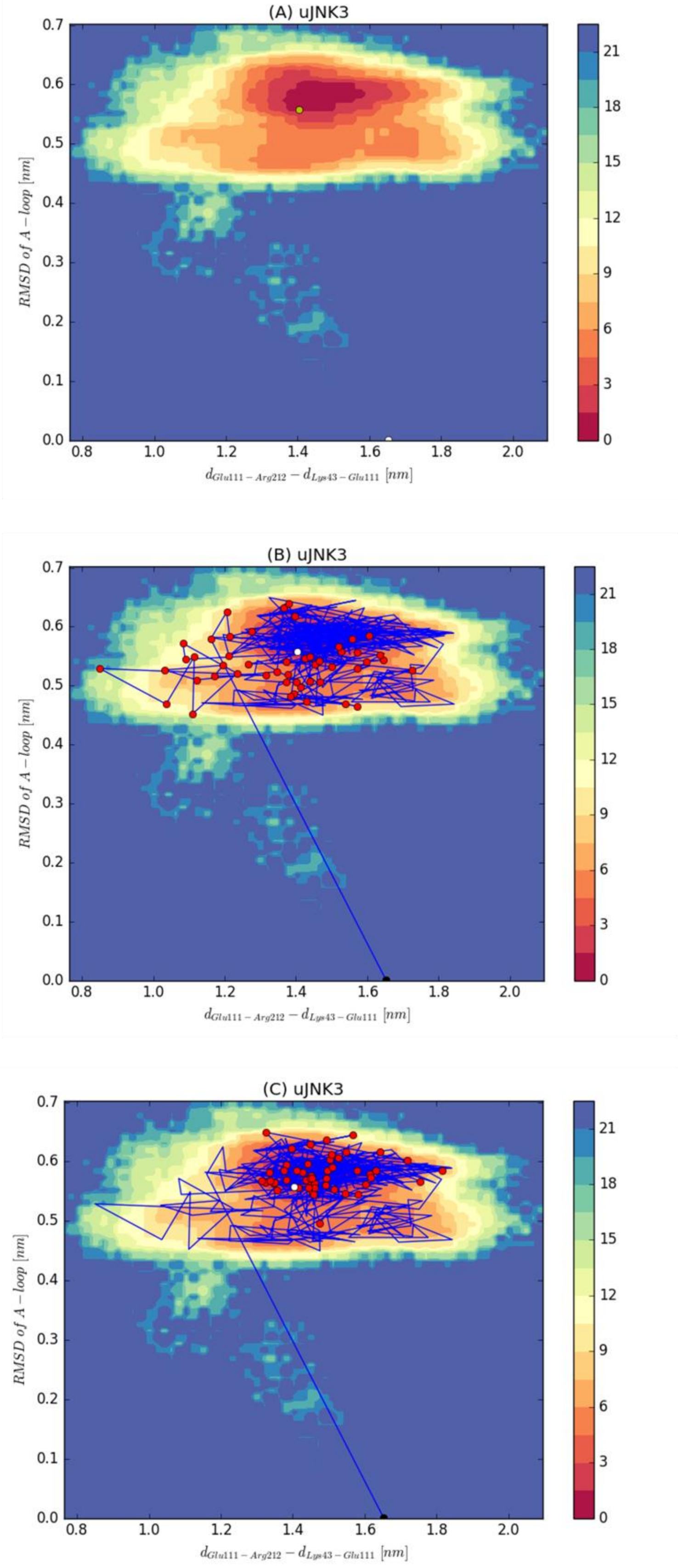

(A) uJNK3
(B) uJNK3
(C) uJNK3
RMSD of A − loop [nm]
$d_{Glu111-Arg212} - d_{Lys43-Glu111}$ [nm]
0.0
0.1
0.2
0.3
0.4
0.5
0.6
0.7
0.8
1.0
1.2
1.4
1.6
1.8
2.0
0
3
6
9
12
15
18
21

Figure S4. The conformational landscape of unphosphorylated JNK3 (uJNK3) kinase. The free energy surfaces are generated as a function of the r.m.s.d of the A-loop and the difference of the distance between Glu111-Arg212 and Lys43-Glu111 residue pairs. A) The free landscape diagram is shown with the start (white dot) and end point (yellow dot) of the simulation. Figure B and C) Illustrates the path travelled by the protein through the timescale of simulation. In Figure B and Figure C, the red dots represents the first and second half of the simulation at an equal interval of 20 ns, repectively.

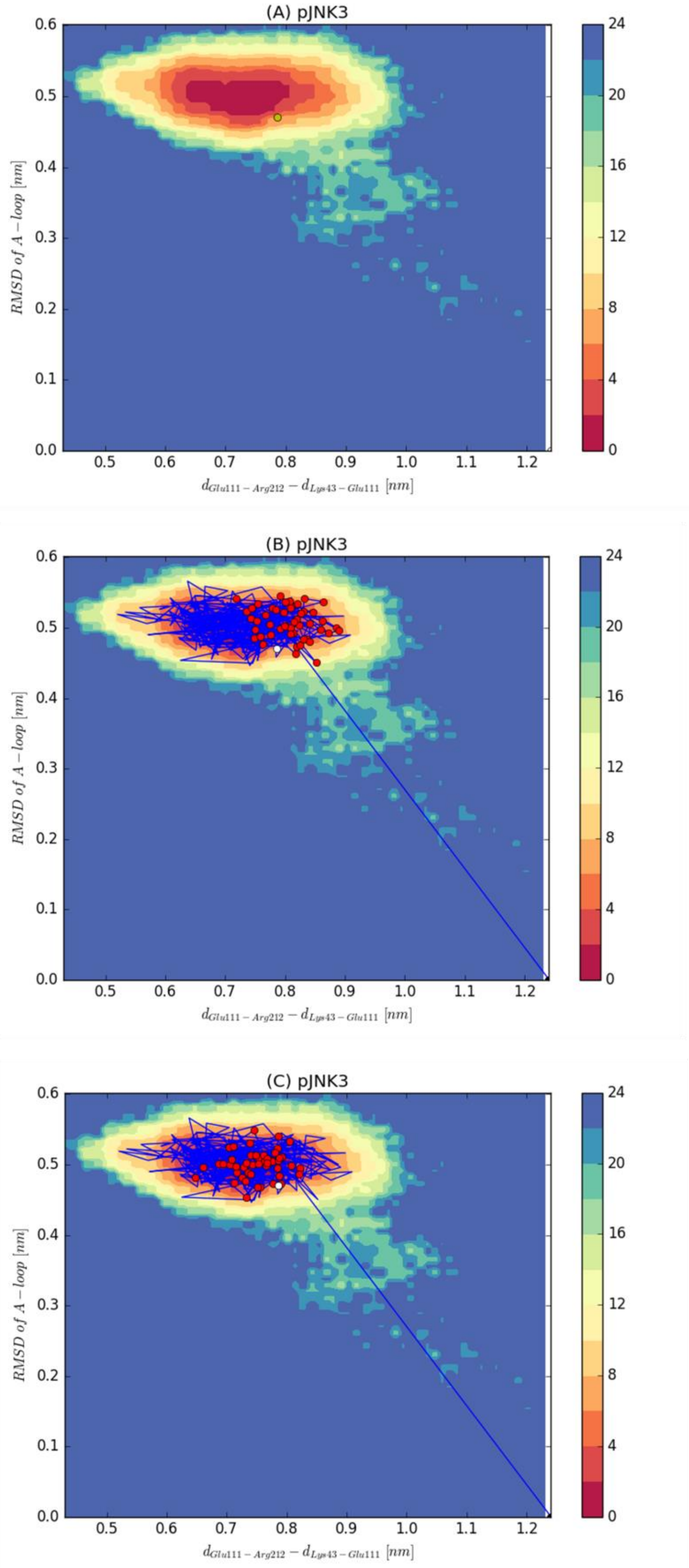

(A) pJNK3
(B) pJNK3
(C) pJNK3
$RMSD\ of\ A-loop\ [nm]$
$d_{Glu111-Arg212} - d_{Lys43-Glu111}\ [nm]$
0.0
0.1
0.2
0.3
0.4
0.5
0.6
0.5
0.6
0.7
0.8
0.9
1.0
1.1
1.2
0
4
8
12
16
20
24

Figure S5. The conformational landscape of phosphorylated JNK3 (pJNK3) kinase. The free energy surfaces are generated as a function of the r.m.s.d of the A-loop and the difference of the distance between Glu111-Arg212 and Lys43-Glu111 residue pairs. A) The free landscape diagram is shown with the start (white dot) and end point (yellow dot) of the simulation. Figure B and C) Illustrates the path travelled by the protein through the timescale of simulation. In Figure B and Figure C, the red dots represents the first and second half of the simulation at an equal interval of 20 ns, repectively.

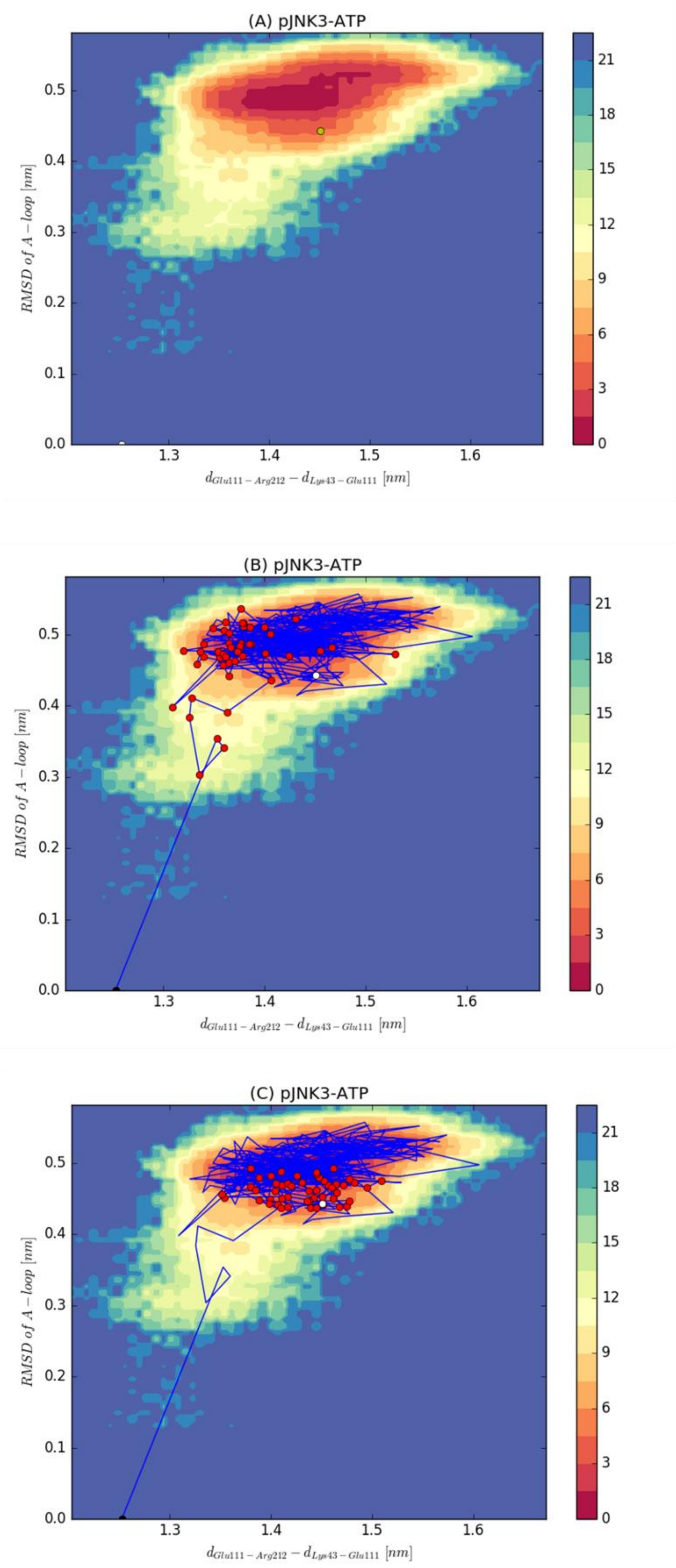
(A) pJNK3-ATP
(B) pJNK3-ATP
(C) pJNK3-ATP
RMSD of A − loop [nm]
d_Glu111 − Arg212 − d_Lys43 − Glu111 [nm]
0.0 0.1 0.2 0.3 0.4 0.5
1.3 1.4 1.5 1.6
0 3 6 9 12 15 18 21

Figure S6. The conformational landscape of ATP-bound phosphorylated JNK3 (pJNK3-ATP) kinase. The free energy surfaces are generated as a function of the r.m.s.d of the A-loop and the difference of the distance between Glu111-Arg212 and Lys43-Glu111 residue pairs. A) The free landscape diagram is shown with the start (white dot) and end point (yellow dot) of the simulation. Figure B and C) Illustrates the path travelled by the protein through the timescale of simulation. In Figure B and Figure C, the red dots represents the first and second half of the simulation at an equal interval of 20 ns, repectively.

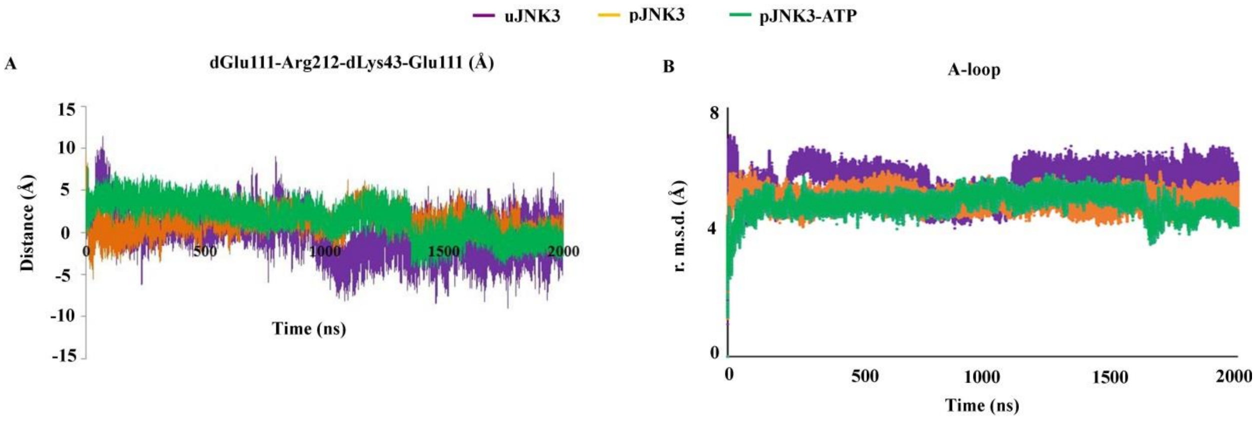

Figure S7. A) The difference of the distance between Glu111-Arg212 and Lys43-Glu111 residue pairs and B) root mean square deviation (r.m.s.d) data of A-loop.

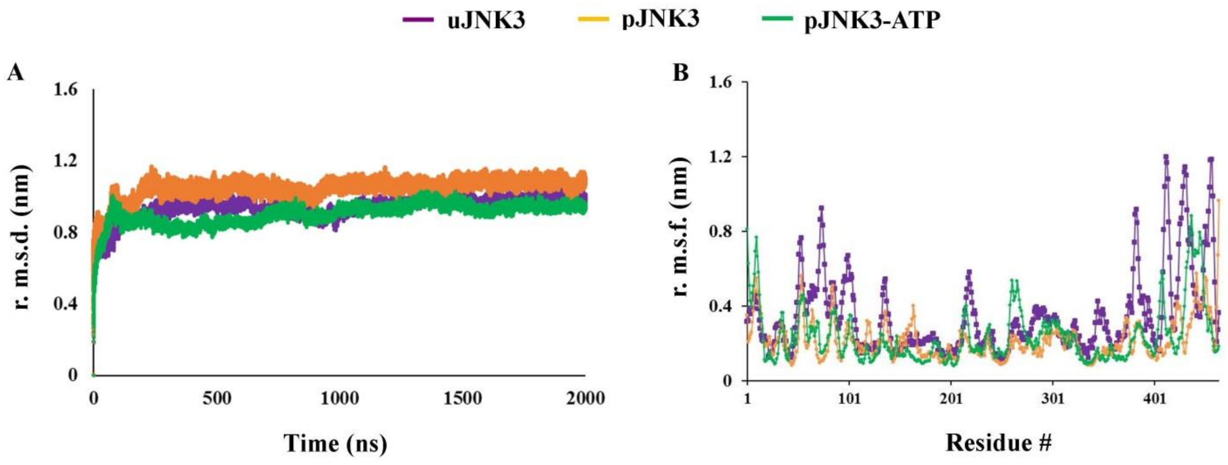

Figure S8. Overall stability of JNK3 kinase during 2 μs MD simulations. A) Cα atoms root mean square deviation (r.m.s.d.) of apo and ATP-bound structures. B) Cα atoms root mean square fluctuations (r.m.s.f.) of apo and ATP-bound structures.

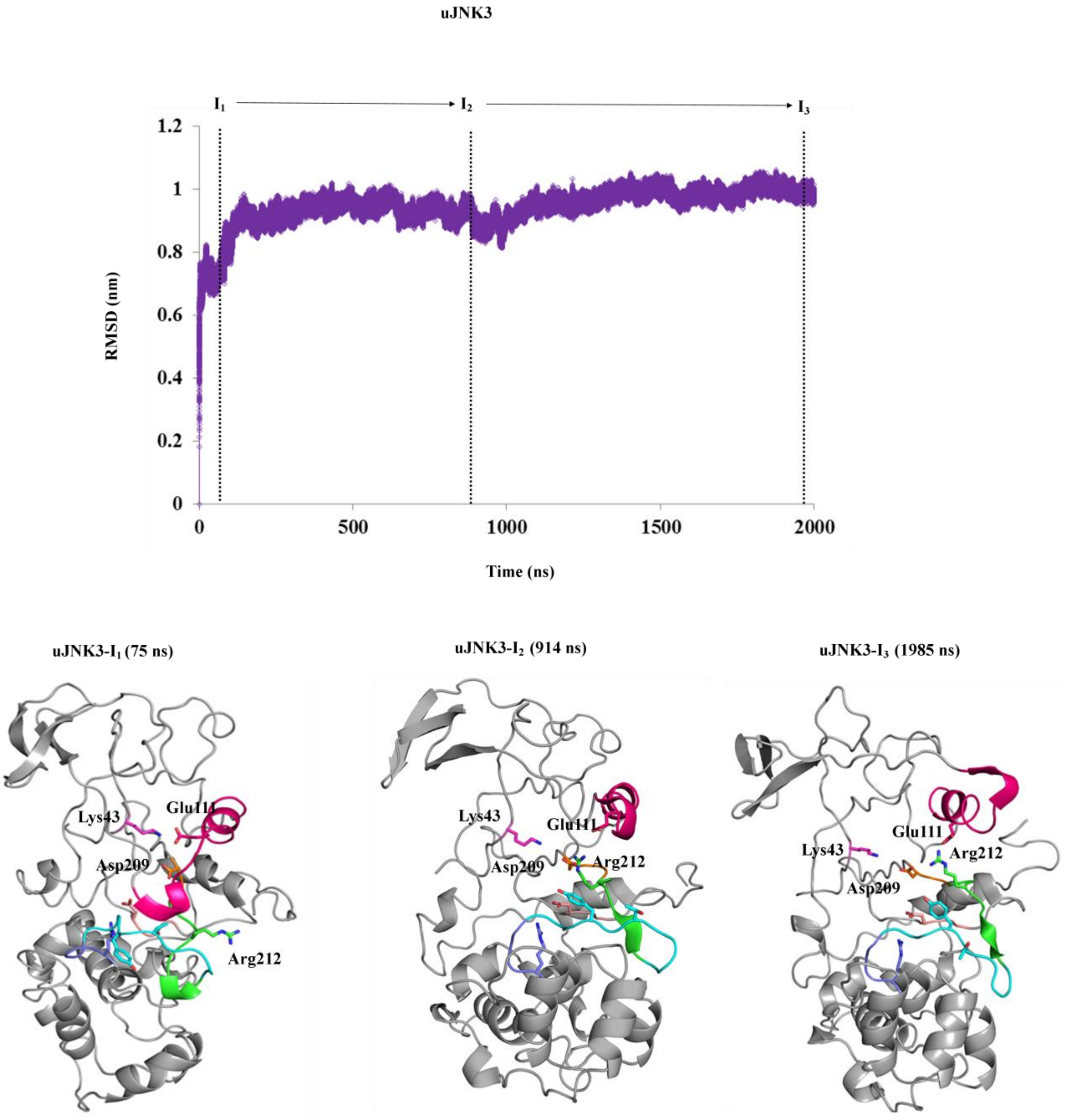

Figure S9. The Cα atoms root mean square deviation (r.m.s.d.) of unphosphorylated JNK3 (uJNK3) kinase with respect to time are shown along with the key intermediate states I_1-I_3.

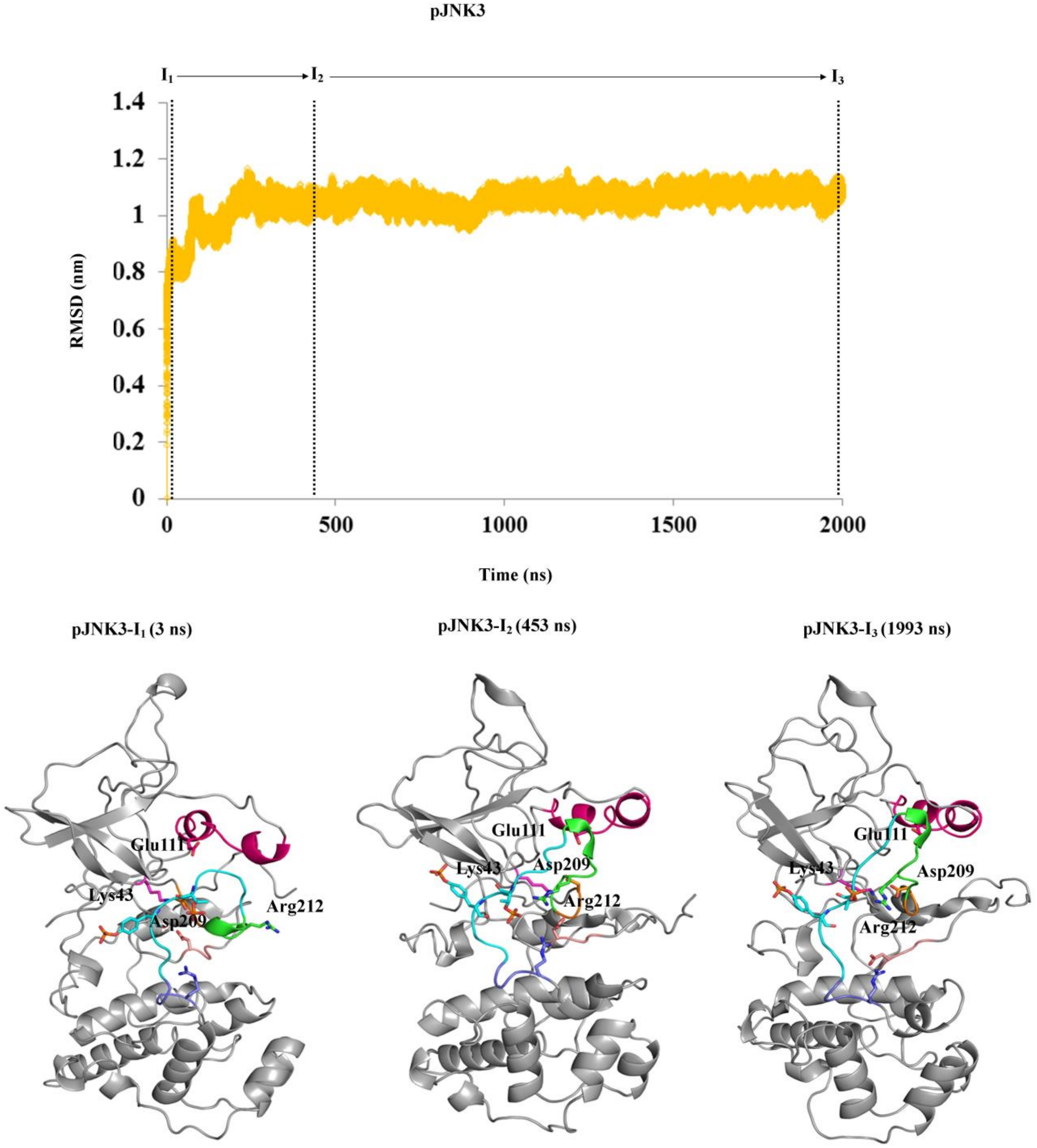

Figure S10. The Cα atoms root mean square deviation (r.m.s.d.) of phosphorylated JNK3 (pJNK3) kinase with respect to time are shown along with the key intermediate states I_1-I_3.

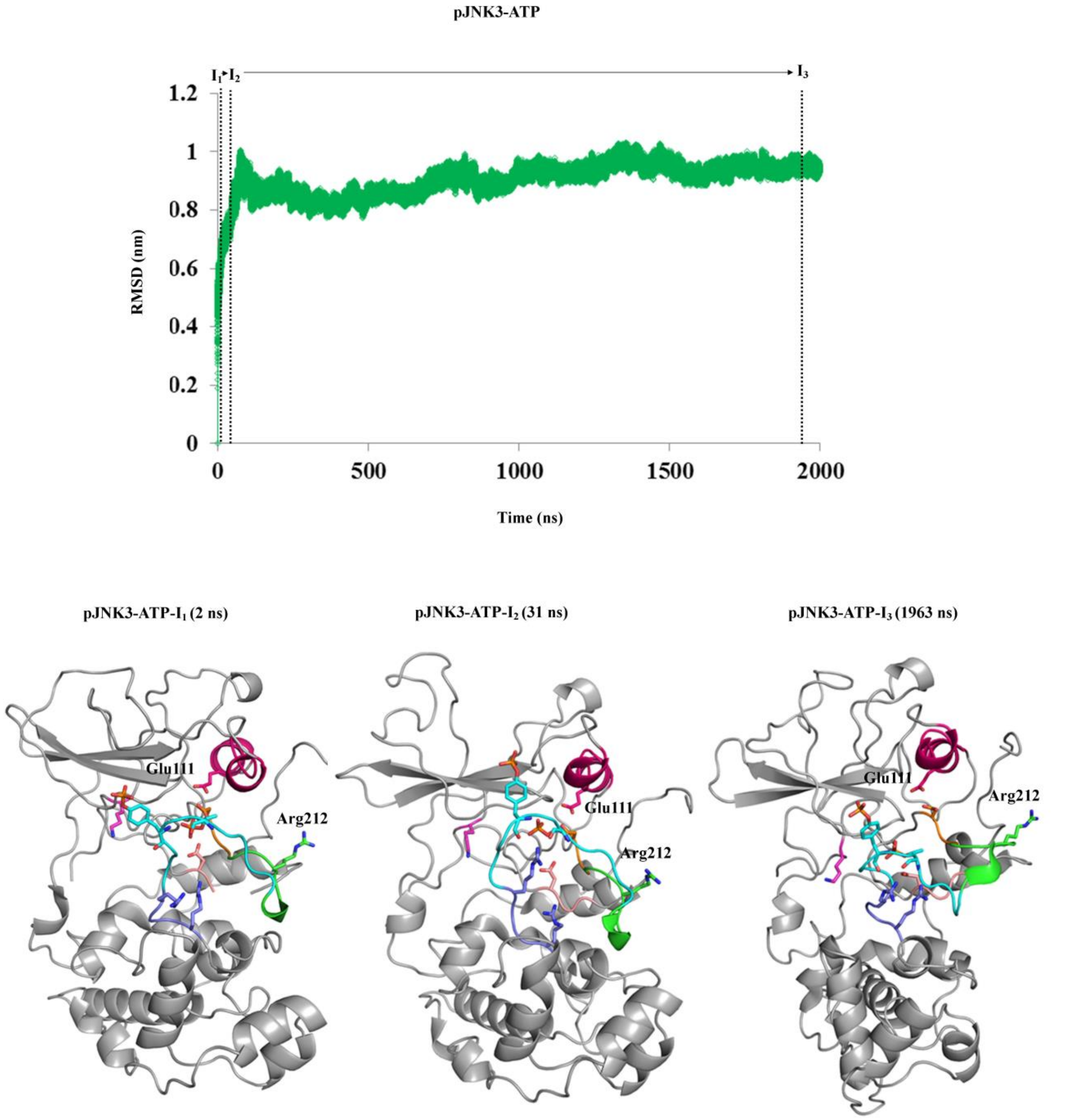

Figure S11. The Cα atoms root mean square deviation (r.m.s.d.) of ATP-bound phosphorylated JNK3 (pJNK3-ATP) kinase with respect to time are shown along with the key intermediate states I_1-I_3.

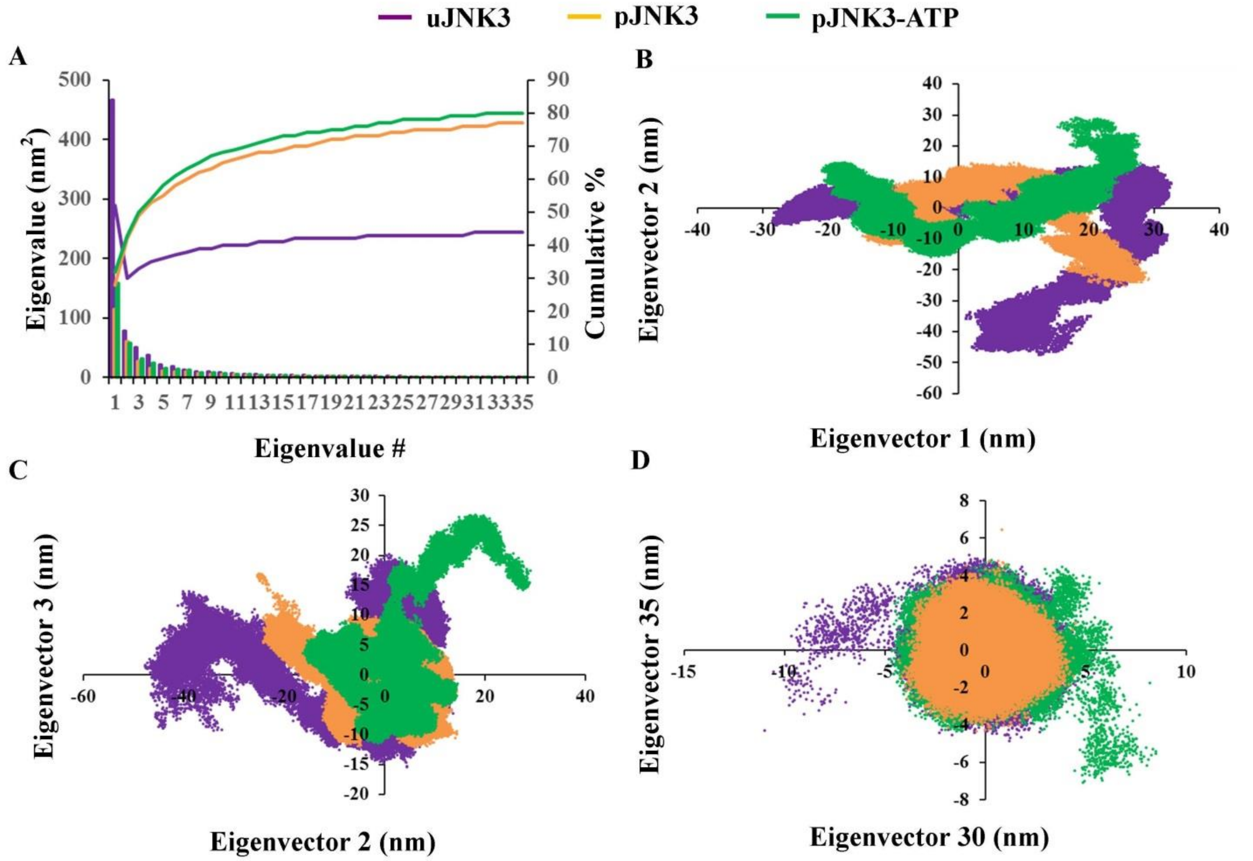

Figure S12. Principal component projections of JNK3 trajectories in phase space. A) The first 35 eigenvectors (bar graph) and their cumulative sum (line graph) of the contribution to the total fluctuations are shown for uJNK3, pJNK3, and pJNK3-ATP. The clouds represent the principal component projection of JNK3 trajectories in phase space; B) eigenvector 1 and 2, C) eigenvector 2 and 3, and D) eigenvector 30 and 35.

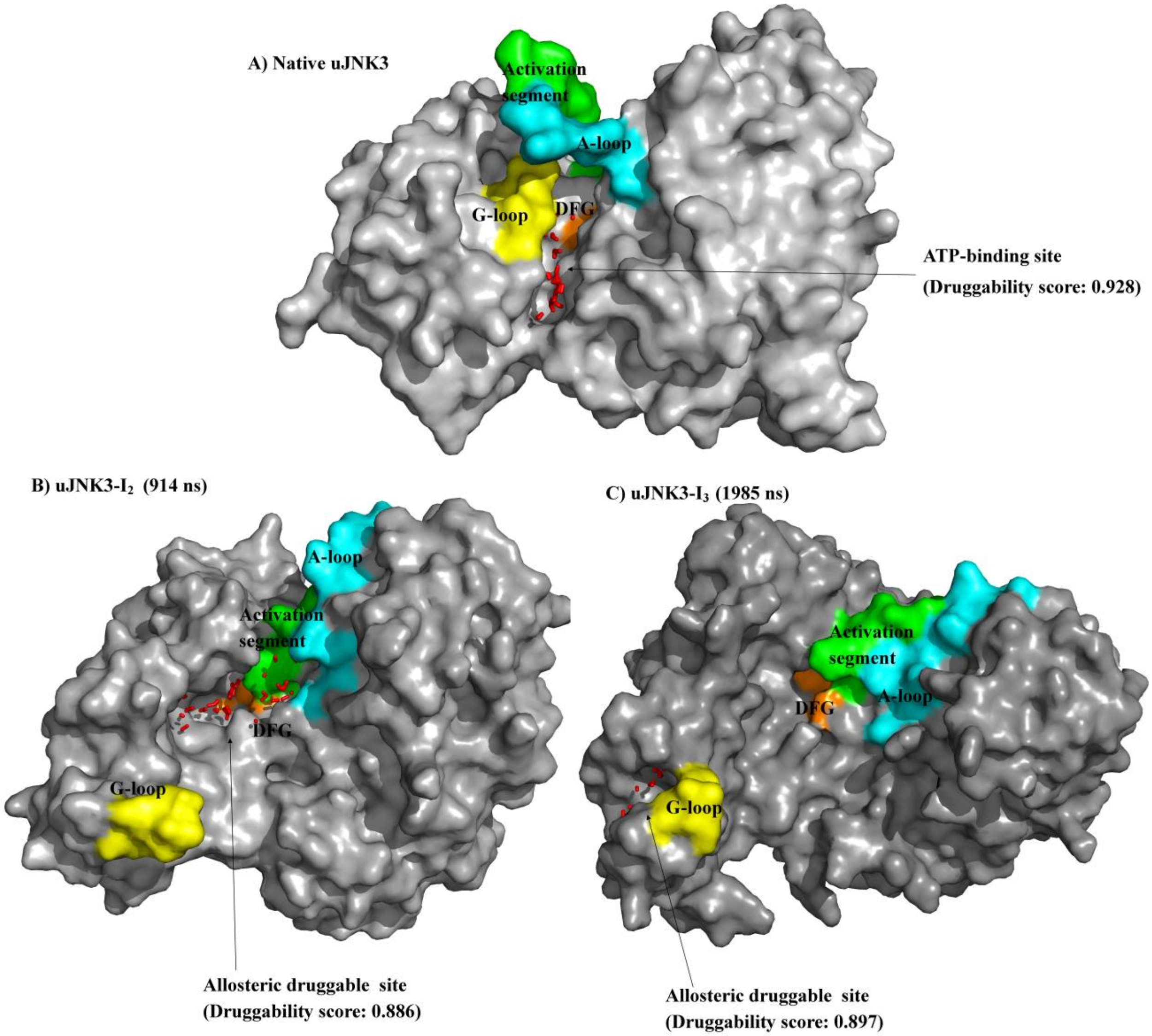

Figure S13. Druggability analysis of the different conformations of uJNK3 indicating allosteric binding pockets. A) The crystal structure of JNK3 (native conformation); B) and C) The intermediate representative conformations of uJNK3 during the simulation timescale of 914 ns and 1985 ns, respectively.

Movie S1: The animation movie for the non-mutated uJNK3. In the non-mutated protein, the representative conformation obtained at step 2 (main manuscript, Figure 4B) was extracted from

the initial trajectory and subjected to another simulation with similar conditions as defined for the original simulation. The highlighted areas are; pink (αC-helix), green (activation segment), orange (DFG motif), cyan (A-loop), purple (P+1 loop), salmon (HRD motif) and gray (N- and C-lobe).

Movie S2: The animation movie for the mutated uJNK3. In the mutated protein, the representative conformation obtained at step 2 (main manuscript, Figure 4B) was extracted from the initial trajectory. The key residue Glu111 was mutated with alanine and subjected to another simulation with similar conditions as defined for the original simulation. The highlighted areas are; pink (αC-helix), green (activation segment), orange (DFG motif), cyan (A-loop), purple (P+1 loop), salmon (HRD motif) and gray (N- and C-lobe).

Animation movie of first and second principal motions

Movies S3-S5: The animation movie for the collective motion of the first eigenvector; uJNK3 (movie S3), pJNK3 (movie S4) and pJNK3-ATP (movie S5). The highlighted areas are; pink (αC-helix), green (activation segment), orange (DFG motif), cyan (A-loop), purple (P+1 loop), salmon (HRD motif) and gray (N- and C-lobe).

Movies S6-S8: The animation movie for the collective motion of the second eigenvector; uJNK3 (movie S6), pJNK3 (movie S7) and pJNK3-ATP (movie S8). The highlighted areas are; pink (αC-helix), green (activation segment), orange (DFG motif), cyan (A-loop), purple (P+1 loop), salmon (HRD motif) and gray (N- and C-lobe).

Reference

Xie, X. *et al.* Crystal structure of JNK3: a kinase implicated in neuronal apoptosis. *Structure.* **6**, 983-991 (1998).
